# Crystal Structure of the Herpesvirus Nuclear Egress Complex Provides Insights into Inner Nuclear Membrane Remodeling

**DOI:** 10.1016/j.celrep.2015.11.008

**Published:** 2015-12-17

**Authors:** Tzviya Zeev-Ben-Mordehai, Marion Weberruß, Michael Lorenz, Juliana Cheleski, Teresa Hellberg, Cathy Whittle, Kamel El Omari, Daven Vasishtan, Kyle C. Dent, Karl Harlos, Kati Franzke, Christoph Hagen, Barbara G. Klupp, Wolfram Antonin, Thomas C. Mettenleiter, Kay Grünewald

**Affiliations:** 1 Division of Structural Biology, Wellcome Trust Centre for Human Genetics, University of Oxford, Roosevelt Drive, Oxford OX3 7BN, UK; 2 Friedrich Miescher Laboratory of the Max Planck Society, 72076 Tübingen, Germany; 3 Institute of Molecular Virology and Cell Biology, Friedrich-Loeffler-Institut, Federal Research Institute for Animal Health, 17493 Greifswald – Insel Riems, Germany

## Abstract

Although nucleo-cytoplasmic transport is typically mediated through nuclear pore complexes, herpesvirus capsids exit the nucleus via a unique vesicular pathway. Together, the conserved herpesvirus proteins pUL31 and pUL34 form the heterodimeric nuclear egress complex (NEC), which, in turn, mediates the formation of tight-fitting membrane vesicles around capsids at the inner nuclear membrane. Here, we present the crystal structure of the pseudorabies virus NEC. The structure revealed that a zinc finger motif in pUL31 and an extensive interaction network between the two proteins stabilize the complex. Comprehensive mutational analyses, characterized both in situ and in vitro, indicated that the interaction network is not redundant but rather complementary. Fitting of the NEC crystal structure into the recently determined cryoEM-derived hexagonal lattice, formed in situ by pUL31 and pUL34, provided details on the molecular basis of NEC coat formation and inner nuclear membrane remodeling.

## Highlights

• Both components of the nuclear egress complex, pUL31 and pUL34, have a unique fold
• pUL31 has a conserved C3H zinc finger motif
• The nuclear egress complex is highly stable with an extensive interaction surface
• Molecular basis of interactions to assemble a curved hexagonal lattice coat is revealed

## Graphical Abstract

**Figure:**
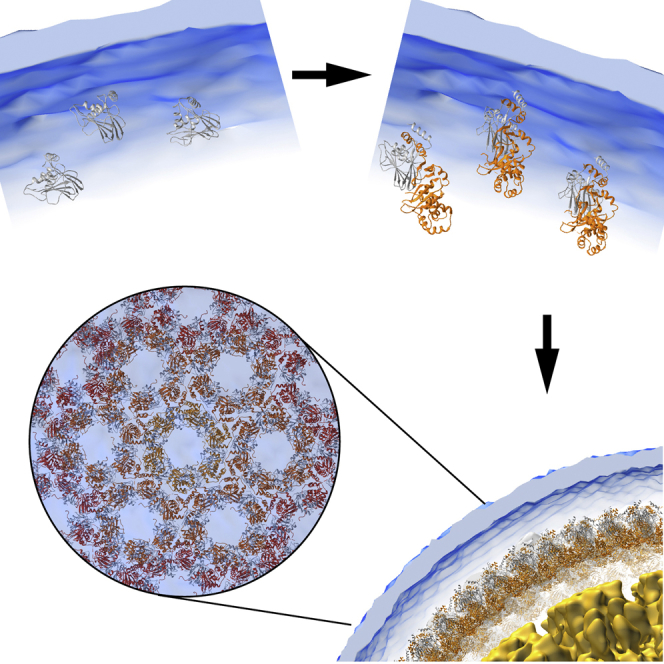

## Teaser

Herpesvirus capsids exit the nucleus by a vesicular transport through the nuclear double membrane. Zeev-Ben-Mordehai et al. present the crystal structure of the coat proteins mediating envelopment of capsids. The structure is used to model the assembly of hexagonal lattice driving vesicle formation at the inner nuclear membrane.

## Introduction

Viruses have developed remarkable strategies to usurp cellular processes for their own needs while maintaining efficient virus production. This is particularly obvious in the life cycle of herpesviruses, which replicate viral DNA and assemble nucleocapsids in the nucleus of non-dividing cells. These capsids are translocated from the nucleoplasm to the cytoplasm via vesicular trafficking through the nuclear envelope, thereby bypassing nuclear pore complexes, the canonical gateways of nucleo-cytoplasmic transport. The translocation of capsids across the nuclear double membrane is a two-step process; first, capsids are enveloped at the inner nuclear membrane, a process referred to as primary envelopment. These enveloped capsids are then fused with the outer nuclear membrane and thereby released into the cytoplasm (for reviews, see Johnson and Baines, 2011, Mettenleiter et al., 2013).

Envelopment of herpesvirus capsids requires extensive restructuring of the host inner nuclear membrane to form capsid-containing vesicles. Budding and scission of membrane vesicles in intracellular trafficking are meditated by dedicated coat proteins, such as clathrin and the COP I and COP II complexes, which induce membrane curvature on the cytosolic membrane face to ultimately extrude cytoplasmic vesicles (McMahon and Gallop, 2005). Vesicle formation in the opposite direction, i.e., away from the cytosol, is less prevalent but does occur. Examples include the invagination of the endosomal membrane during the formation of multi-vesicular bodies and the egress of HIV and other enveloped viruses at the plasma membrane, as mediated by the endosomal sorting complexes required for transport (ESCRT) machinery (Votteler and Sundquist, 2013). Herpesvirus vesicle formation into the nuclear envelope lumen belongs to this latter category, because it is directed away from the nucleoplasm, which is topologically equivalent to the cytoplasm.

It is well accepted that herpesvirus capsid nuclear egress is mediated by two conserved herpesvirus proteins, designated as pUL31 and pUL34, in the alphaherpesviruses herpes simplex virus 1 (HSV1) and pseudorabies virus (PrV) (Johnson and Baines, 2011, Mettenleiter et al., 2013). pUL34 is a type II single-pass transmembrane protein residing in the endoplasmic reticulum and nuclear membranes, while pUL31 is a soluble protein targeted to the nucleoplasm by a nuclear localization signal. In the nucleus of infected cells, pUL34 recruits pUL31 to the inner nuclear membrane to form the nuclear egress complex (NEC). Both proteins are required for the transport of viral capsids through the nuclear envelope and, thus, for productive herpesvirus replication (Bubeck et al., 2004, Fuchs et al., 2002, Klupp et al., 2000, Reynolds et al., 2001). Although the participation of other viral and cellular proteins in perinuclear vesicle formation during infection cannot be excluded, transient or stable co-expression of both proteins in eukaryotic cells results in the generation of periplasmic vesicles originating from the inner nuclear membrane, even in the absence of capsids (Klupp et al., 2007). Moreover, pUL31 and pUL34 are sufficient for budding of small vesicles and scission into giant unilamellar vesicles (GUVs) (Bigalke et al., 2014, Lorenz et al., 2015). Furthermore, in this minimal model membrane system, artificial tethering of pUL31 to GUV membranes induced its self-oligomerization, which resulted in vesicle budding and scission into the GUV lumen (Lorenz et al., 2015). Thus, the NEC represents self-sufficient machinery that can mediate inwardly directed vesicle formation.

Here, we present the crystal structure of the PrV NEC, revealing that both components have a unique fold and that pUL31 contains a zinc finger motif. The structure, with its extensive interaction surface, explained the complex’s high stability. The crystal structure was further fitted into the recently determined cryoelectron microscopy (cryo-EM) hexagonal lattice formed in situ by pUL31 and pUL34 (Hagen et al., 2015), providing details on the intra- and inter-hexamer interactions leading to formation of the NEC coat.

## Results and Discussion

### Structure Determination

To produce high-quality diffracting crystals of the PrV NEC, a pUL31 construct (amino acids [aa] 26–271) lacking the nuclear localization signal was co-expressed in *E. coli*, together with the conserved region of pUL34 (aa 1–179). Data were collected for a selenomethionine derivative of the complex, the electron density map was calculated from experimental phasing, and the structure was refined to a 2.9 Å resolution (see Table S1 for the statistics for diffraction data collection and refinement of the atomic model). One heterodimer was present in the asymmetric unit. For pUL31, the complete aa chain was traced, with the exception of disordered loops at positions 158–159, 173–174, and 224–228 and residue 101. For pUL34, the chain was traced from aa 4 to 174, with the exception of a disordered loop at positions 23–25. The complex structure is overall ordered, with helices α5 and α10 of pUL31 being the most mobile parts of the complex.

### Both pUL31 and pUL34 Have a Unique Fold

**Figure 1:**
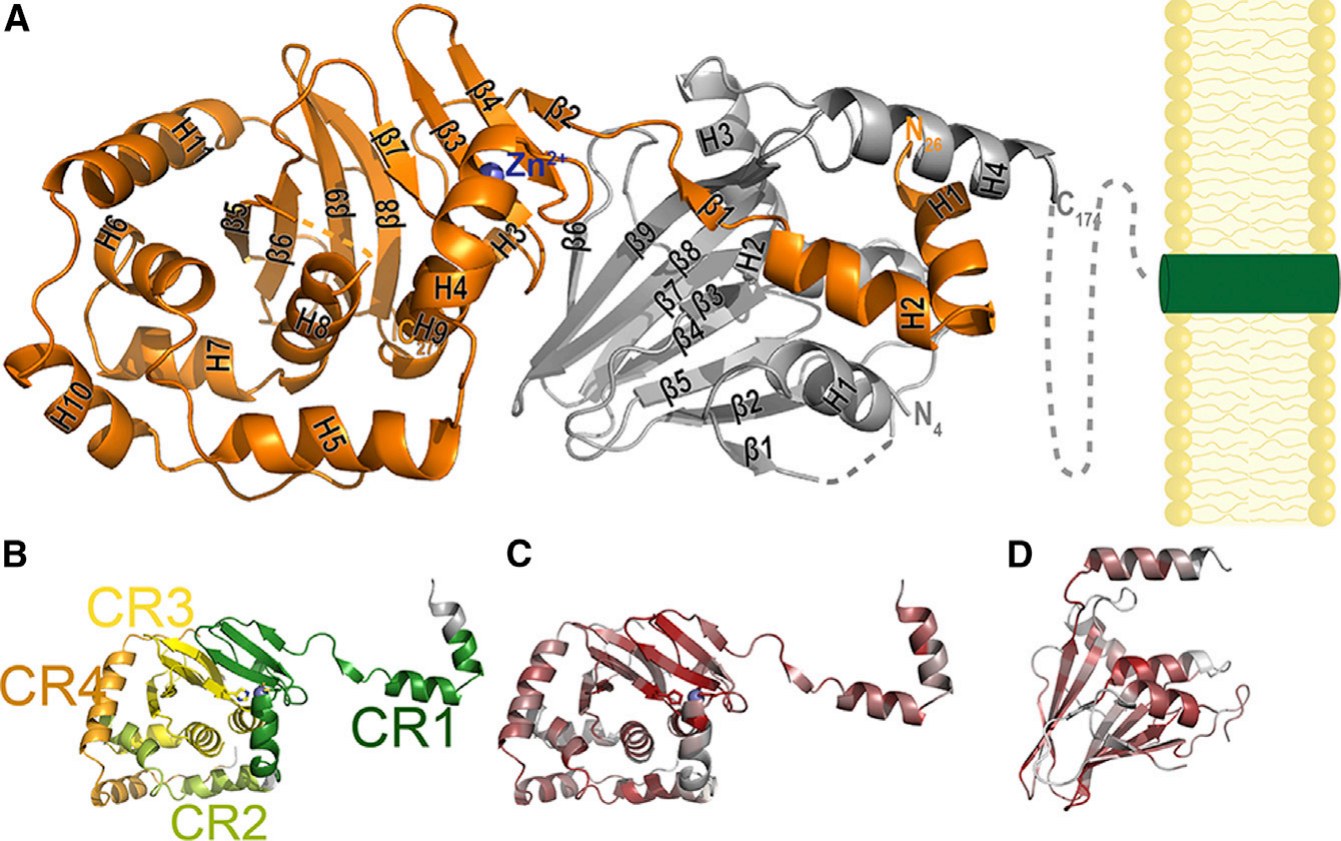
Structure and Conservation of the NEC (A) The pUL31-pUL34 complex in cartoon representation. pUL31 is shown in orange, and pUL34 is shown in gray. Unresolved regions are indicated by dashed lines, and the pUL31 bound Zn^2+^ ion is indicated in blue. Secondary structure elements are labeled on the structure (see also Figure S2). The inner nuclear membrane (yellow) and the transmembrane region of pUL34 (green) are schematically drawn. (B) pUL31 structure colored according to the previously described conserved regions CR1–CR4 (Lötzerich et al., 2006). CR1, dark green; CR2, light green; CR3, yellow; CR4, orange. The Zn^2+^ ion and its complexing residues, shown in stick representation, are indicated. (C) Mapping of the sequence conservation for pUL31 (see sequence alignment in Figure S2A), ranging from dark red for the most conserved to light gray for the least conserved residues. (D) Mapping of the sequence conservation for pUL34 (see sequence alignment in Figure S2B). Color code is as in (C). See also Figure S2.

The crystal structure of the heterodimer displays an elongated shape of ∼50 Å in width and ∼100 Å in length, with the putative membrane-distal end being composed of pUL31 and the membrane-proximal part being composed mainly of pUL34 (Figure 1). This shape is highly similar to that of the heterodimer determined in solution by small-angle X-ray scattering (Hagen et al., 2015). Each protein of the heterodimer is, overall, globular, with the exception of helices α1 and α2 of pUL31, which extend around pUL34. The topology of both proteins seems to be unique (Figure S1), and no structural homologs could be found in the protein database (based on Dali server searches against the PDB).

pUL34 has a β sandwich fold comprising nine β strands. Strands β3, β6, β8, and β9 form one sheet of the fold that faces pUL31, and strands β1, β2, β4, β5, and β7 form the other sheet. Four helices cap the fold on the membrane-proximal side. Both the N and the C termini of pUL34 are located on the membrane-proximal side of the molecule (Figure 1A).

pUL31 has a complex topology (Figure 1; Figure S1), with 11 helices and nine β strands. Its core is made of helices α6–α8 and strands β5–β9 and is surrounded by four helices (α4–α5 and α10–α11). Coloring the protein according to the previously defined conserved regions (CR) 1 to 4 (Lötzerich et al., 2006) confirmed that CR1 is mainly involved in interaction with pUL34, while CR3 represents the structural core (Figure 1B). Notably, a C3H zinc finger (ZNF) motif is present, with three cysteines contributed by CR1 and a histidine by CR3. Mapping of conserved aa onto the structure revealed conservation of further sites and structural elements for each protein (Figures 1C and 1D; Figure S2). A subsequent detailed analysis of the structure highlighted the nature of these specific sites and motives.

### pUL31 ZNF Motif Is Crucial for the Heterodimer Formation and Function

**Figure 2:**
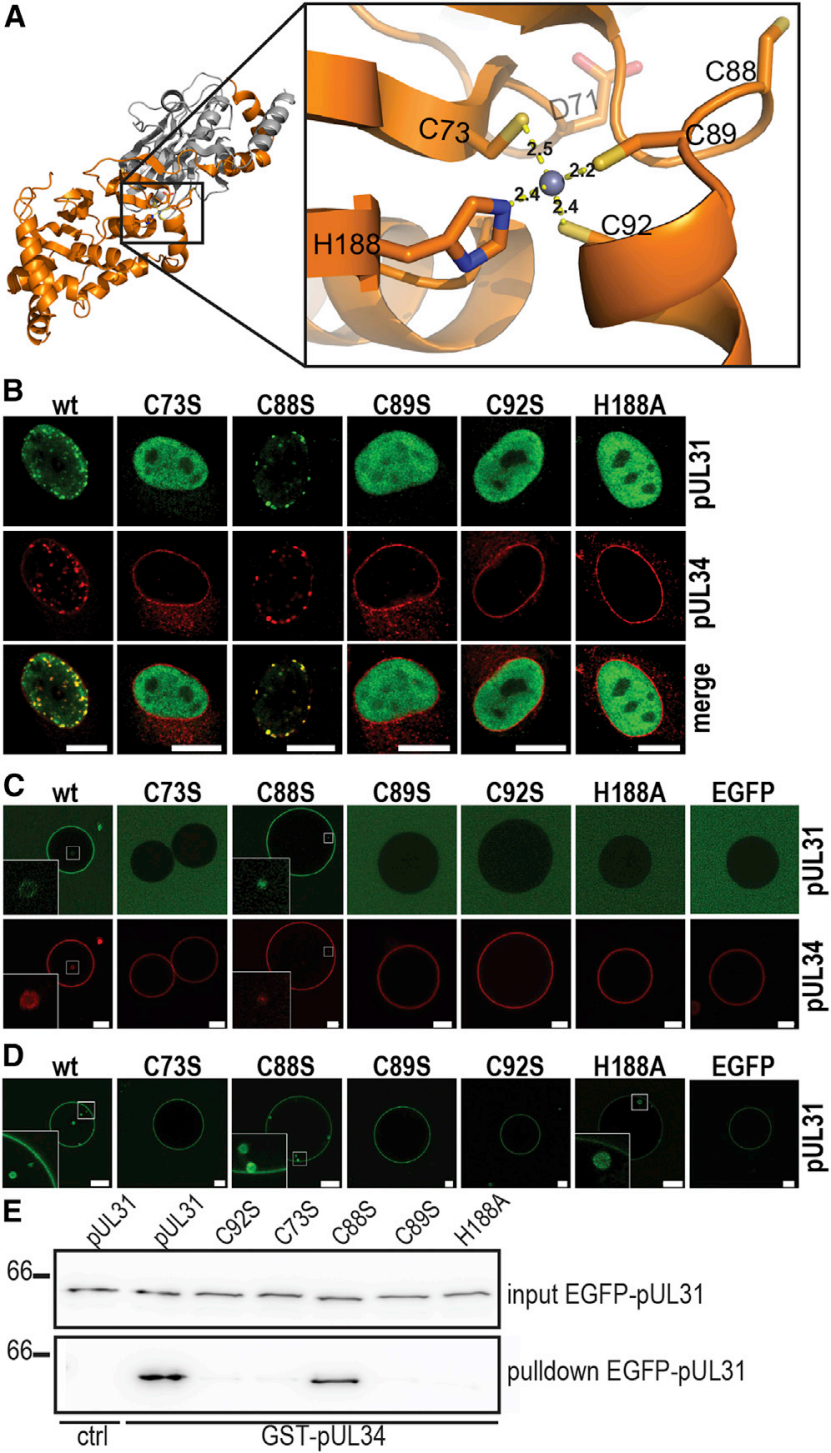
pUL31 ZNF Motif Is Vital for NEC Function (A) Close-up view on the pUL31 ZNF motif. The side chains Cys73, Cys89, Cys92, and His188 coordinating the Zn^2+^ ion are shown as sticks, and respective distances (in angstroms) are indicated. Colors are as described in Figure 1A. (B) In situ phenotype of pUL31 mutants. RK13 cells were co-transfected with WT pUL34 and pUL31 WT or mutant constructs. Proteins were detected by immunofluorescence with respective antibodies followed by confocal microscopy of the nuclear region. Anti-pUL31 is shown in green; anti-pUL34 is shown in red. Scale bars, 10 μm. (C and D) In vitro phenotype of mutations in the ZNF motif affecting vesicle formation. (C) Soluble WT pUL31 (expressed as an EGFP fusion) was added to pUL34-GUVs. Recruitment of pUL31 and vesicle formation into the GUV lumen was assayed. Green channel shows pUL31-EGFP; red channel shows Alexa Fluor 546-labeled pUL34. (D) His_6_-tagged-EGFP or His_6_-tagged-EGFP-pUL31 proteins were directly tethered to Ni-NTA-DGS containing GUVs, in the absence of pUL34. Scale bars, 10 μm. (E) Western blot of pull-downs showing pUL31-pUL34 interactions. Anti-EGFP antibodies were used to detect pull-downs using GST (ctrl) or GST-pUL34 as baits and EGFP-pUL31 WT and mutants as prey (for details, see Experimental Procedures).

The four residues coordinating the Zn^2+^ ion (C73, C89, C92, and H188) belong to the most conserved residues in pUL31 (Figure 1C; Figure S2). ZNFs are important compact structural motifs that are known to function as interaction modules (Kochańczyk et al., 2015). The ZNF motif in pUL31 is on the surface facing pUL34 (Figure 2A). To define the function of the ZNF motif in pUL31, cysteines C73, C89, and C92 were individually replaced by serines, and the histidine H188 was replaced by an alanine, and functional analyses of the mutant proteins were performed both in vitro with artificial membranes and in situ in eukaryotic cells. In cells co-expressing the two proteins, wild-type (*wt*) pUL31 is recruited to the inner nuclear membrane by the membrane-anchored pUL34, and characteristic “speckles” were observed. pUL31 mutants defective in binding of pUL34 have been shown to mis-localize diffusely to the nucleoplasm (Klupp et al., 2007). To assess the impact of the ZNF residues on the function of pUL31, each of the ZNF mutants, as well as a serine substitution of the adjacent (C88S), was co-expressed with wild-type (WT) pUL34 in rabbit kidney (RK13) cells. While the pUL31 C88S mutant co-localized with pUL34 at the nuclear rim and showed characteristic WT-like “speckles,” all ZNF mutants exhibited diffuse nucleoplasmic localization (Figure 2B; Table S2), indicating a defect in interaction.

To further define whether the ZNF mutants directly affect the membrane deformation activity of pUL31, we used the recently established in vitro system in which full-length pUL34 is reconstituted into artificial membranes; i.e., GUVs (Lorenz et al., 2015). Addition of soluble WT pUL31 (expressed as an EGFP-fusion protein) to GUVs containing pUL34 (pUL34-GUVs) resulted in the recruitment of the protein to the GUV surface and induced vesicle budding and scission into the GUV lumen, a process that is topologically identical to the NEC-mediated vesicle formation at the nuclear envelope (Figure 2C). In contrast to WT pUL31, all four ZNF mutants failed to bind pUL34-GUV membranes or induce vesicle formation. Consistent with the results from the cellular localization assay, the C88S mutant did not affect binding to pUL34-GUVs and induced WT pUL31-like formation of intra-GUV vesicles.

The failure of pUL31 mutants to induce vesicle formation in pUL34 GUVs could be caused by defects in their membrane remodeling activity and/or in their ability to bind pUL34, possibly as a result of severe misfolding of the protein. To distinguish between the two possibilities, the ability of pUL31 mutants to bind pUL34 was analyzed by a GST (glutathione S-transferase)-pull-down assay, using pUL34 lacking the transmembrane region as bait. All four pUL31 ZNF mutants failed to bind pUL34, while the C88S mutant behaved like WT (Figure 2E; Table S2). This is in agreement with the cellular localization assay and the pUL34-GUV in vitro data and confirms that lack of interaction caused the observed phenotypes.

To test whether the ZNF residues not only affect the interaction with pUL34 but also the membrane remodeling activity of pUL31, WT pUL31 and the mutant proteins were each directly tethered to GUV membranes, bypassing the need for pUL34 for membrane anchorage (for details, see Supplemental Experimental Procedures and Lorenz et al., 2015). Recruitment of WT pUL31 and the C88S mutant resulted in intra-GUV vesicle formation. In contrast, proteins mutated in C73, C89, or C92 were defective in membrane remodeling. Interestingly, the H188A mutant was able to induce vesicle formation, but to a lower extent than WT (Figure 2D; Table S2).

PrV pUL31 mutants defective in nuclear envelope vesicle formation do not support herpesvirus replication (Paßvogel et al., 2015). Indeed, only WT pUL31 and mutant pUL31 C88S showed full complementation, i.e., viral titers similar to WT PrV in a trans-complementation assay of pUL31-deleted PrV on RK13 cells stably expressing the mutated proteins (Table S2). In contrast, all four pUL31 ZNF mutants could not functionally substitute for WT pUL31. Taken together, these results indicate that the ZNF motif is indispensable for NEC formation and the membrane remodeling necessary for the budding and scission of vesicles.

### The NEC Is a Very Stable Complex with an Extensive Interaction Surface

**Figure 3:**
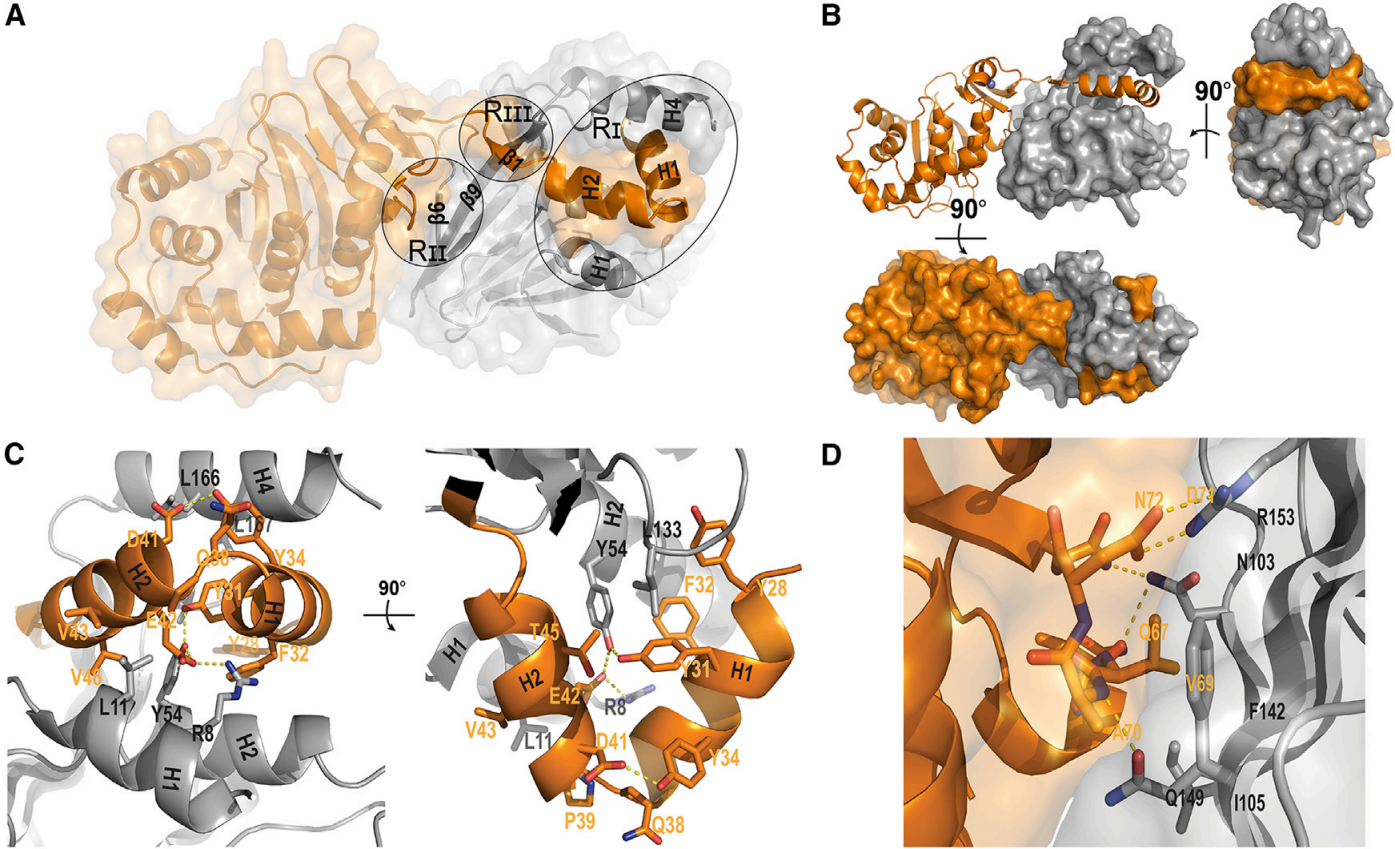
The NEC Has an Extensive Interaction Surface that Can Be Divided into Three Regions (A) Overview of the three interaction regions, marked Rɪ–Rɪɪɪ. Colors are as in Figure 1A. (B) Three orthogonal views of the complex highlighting the extent of the pUL31 N-terminal “arm.” (C and D) Close-up views on regions ɪ and ɪɪ, respectively. Residues involved in the interaction are shown in sticks; hydrogen bonds are indicated by yellow dotted lines.

All attempts to dissociate the purified complex with high salt and mild detergents were unsuccessful (data not shown). While high-level expression of a pUL34 (aa 1–179) fragment by itself was successful, pUL31 could not be efficiently overexpressed alone, suggesting that pUL31 is not stable in the absence of pUL34. In contrast, the heterodimer proved to be very stable, as measured by circular dichroism, giving a high melting temperature of 57°C (Figure S3). The extensive buried surface area on both proteins (∼1,850 Å^2^, corresponding to ∼13%, for pUL31; and ∼1,750 Å^2^, corresponding to 18%, for pUL34) is likely the cause of the observed high stability of the complex. The two proteins have specific intermolecular hydrogen bonds in three regions that are further enforced by hydrophobic interactions (Figure 3). These three regions were analyzed in more detail.

### Region ɪ: Extended pUL31 Angular “Arm” Fitting into a pUL34 Groove

The most striking interacting region, region ɪ (Rɪ), is that of a long N-terminal pUL31 angular “arm” formed by helices α1 and α2, that fits tightly into a specific groove in pUL34 (Figures 1 and 3B). The ∼80° angle between the helices that make the arm reach around pUL34 in a “hugging” fashion is stabilized by hydrogen bonds between the side chains of Y34 and D41 (Figure 3B). The interaction of pUL31 with pUL34 in this region is mainly restricted to three residues on pUL34 helices α1 and α2, viz., pUL34 R8 and L11 on helix α1 and Y54 on helix α2. pUL34 Y54 is key for this interaction region, forming hydrogen bonds with both pUL31 Y31 and E42. Interaction with pUL34 helix α4, forming the other side of the groove, is mainly hydrophobic and mediated through L167. A hydrophobic pocket is formed by residues from both pUL34 and pUL31, particularly pUL34 L133 and pUL31 F32 (Figure 3C). The importance of these residues for the interaction was verified by site-specific mutagenesis. Mutations in pUL31 at position Y31 abolished pUL34 binding, whereas mutating position Y34 strongly reduced the interaction as evident in GST pull-down assays (Figure S5; Table S2). Consistently, both of these pUL31 mutants failed to bind to pUL34-GUVs (Figure S4; Table S2). As indicated, Y54 of pUL34 is key for the interaction in Rɪ, and, accordingly, mutating this residue abolished pUL31 binding (Figure S4; Table S2). Mutating L167 to an alanine strongly reduced pUL31 binding. Both pUL34 mutants also failed to recruit WT pUL31 either completely (pUL34 Y54A) or recruited it less efficiently (L167A) when reconstituted into GUVs (Figure S4; Table S2). Accordingly, pUL31 Y31A was not recruited to the nuclear envelope by WT pUL34 in co-transfected cells, and pUL34 Y54A failed to recruit WT pUL31 (Figure S4; Table S2). Both mutant proteins were also unable to complement the absence of the respective WT protein in virus-infected cells (Table S2). The pUL31 Y34A and pUL34 L167A mutations showed, in this respect, a less severe phenotype. In agreement with its reduced but not abolished capability, pUL31 Y34A was still recruited to the nuclear envelope in the presence of pUL34. Similarly, pUL34 L167A can recruit pUL31 to the nuclear envelope, and both proteins can replace the corresponding WT protein in complementation assays, although less efficiently.

Notably, all the residues involved in Rɪ are highly conserved (Figures 1C and 1D; Figure S2), consistent with their crucial function in the interaction of both proteins. The very tight fit of the pUL31 arm into the pUL34 groove ensures a unique specificity and avidity for the interaction between the NEC partners (Movies S1 and S2).

### Region ɪɪ: Interaction Surface in the Core of the Complex

Region ɪɪ (Rɪɪ) is formed by the pUL31 loop connecting helix α3 and β3 comprising residues 67–72 and pUL34 residues from β6 and β9 (Figure 3D). pUL31 residue D71 forms a strong salt bridge with two hydrogen bonds to pUL34 R153 in pUL34 β9 (Figure 3D). N103 from pUL34 β6 has side chain and backbone interactions with pUL31 N72 and Q67, respectively. This region also exhibits hydrophobic interactions between pUL34 V69 and F142 from pUL34 β9 and I105 from β6.

The importance of the D71-R153 salt bridge for heterodimer formation was validated by site-specific mutagenesis. pUL31 D71 was substituted to an arginine (D71R), and pUL34 R153 was mutated to aspartic acid (R153D) and assayed by GST pull-down. Mutation of either of these residues abolished the interaction with the respective WT counterpart (Figure S4; Table S2). Consistent with this, pUL31 D71R was not recruited to pUL34-GUVs and failed to induce vesicle formation (Figure S4; Table S2). Similarly, WT pUL31 did not bind to GUVs that displayed reconstituted pUL34 R153D (Figure S4; Table S2). Interestingly, pUL31 D71R was able to induce vesicle formation if directly tethered to GUVs, indicating that the mutation does not affect the pUL31 membrane remodeling activity but specifically inhibits pUL34 binding (Figure S4; Table S2). Consistent with these results, pUL31 D71R was not recruited to the nuclear envelope by WT pUL34 in co-transfected cells. pUL34 R153D, despite showing a nuclear envelope staining, could not recruit WT pUL31 to the nuclear envelope and was also unable to complement the defect of the deletion mutant (Figure S4; Table S2). Mutating pUL34 phenylalanine at position 142 to alanine (F142A) resulted in a severe interaction phenotype, but not to the same extent as mutating the D71-R153 salt bridge. pUL34 F142A reconstituted into GUVs failed to recruit WT pUL31; hence, no vesicle formation was detectable (Figure S4; Table S2). In the GST pull-down assay, binding of pUL31 was reduced to about 20% of WT levels (Figure S5; Table S2). Whereas pUL34 F142A localized to the nuclear rim of transfected cells, the recruitment of WT pUL31 was impaired but not abolished, which is consistent with the remaining interaction level observed in GST-pull-downs (Figure S5; Table S2). Together, the interactions in Rɪɪ form a central interaction surface. Notably, pUL31 residue D71 is in very close proximity to the ZNF motif, particularly C73 (Figure 2A); thus, the structural integrity ensured by the ZNF motif directly affects the interactions in Rɪɪ.

### Region ɪɪɪ: Crossover between pUL31 and pUL34 Chains

**Figure 4:**
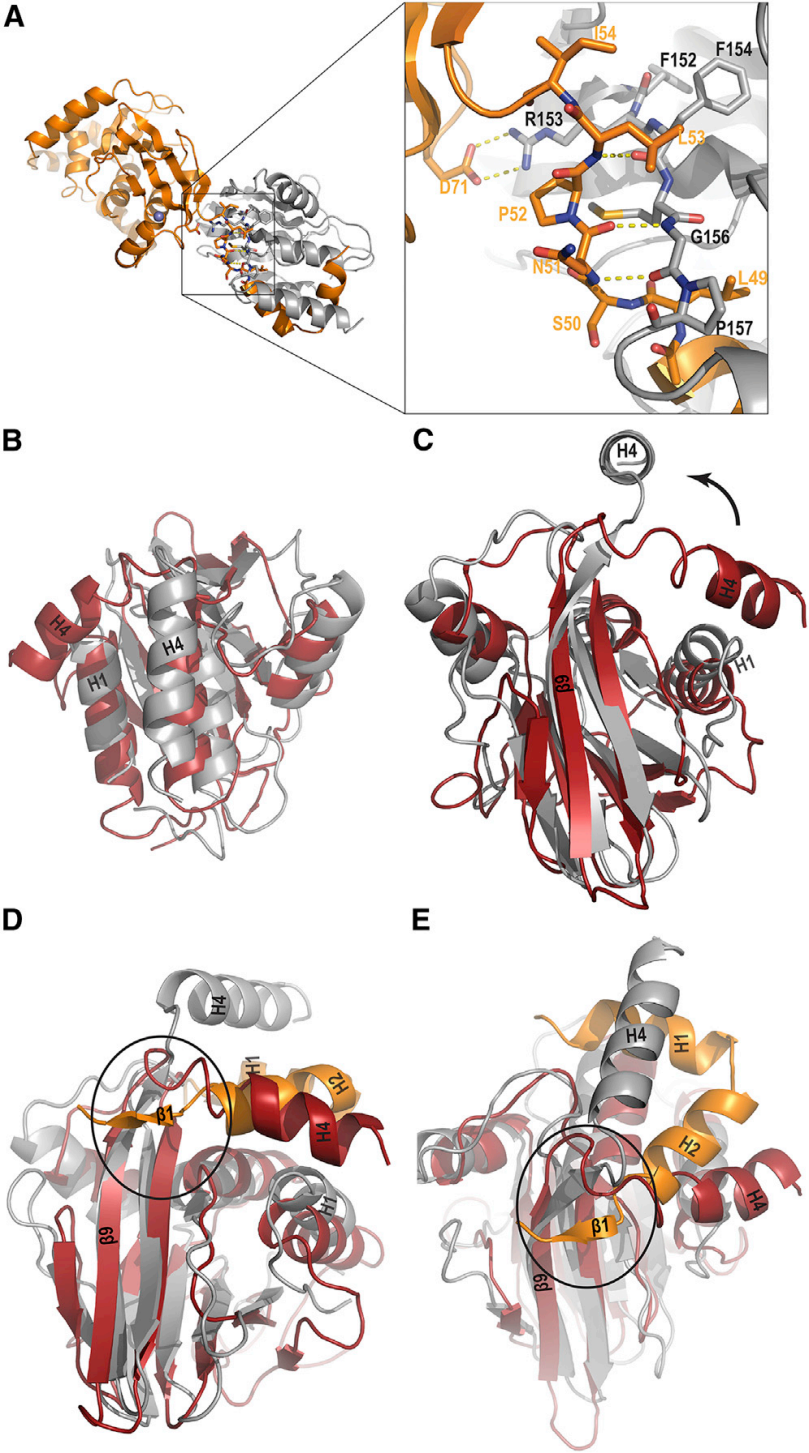
Binding of pUL31 Induces a Major Conformational Change in pUL34 (A) Residues involved in Rɪɪɪ are shown in sticks, and hydrogen bonds are indicated by dotted lines. Colors as in Figure 1A. (B–E) Superposition of the mouse CMV pUL34 homolog M50 (PDB: 5A3G) on the complex. (B and C) Two orthogonal views of the superposition showing M50 in red and pUL34 within the NEC structure in gray. (D and E) Two views of the superposition that include pUL31 helix α1, α2, and β1 in orange. Ovals indicate region Rɪɪɪ.

A third interacting region (Rɪɪɪ) is formed by interaction of the end of the very long pUL34 β9 strand (aa 154–157) with the short pUL31 β1 strand (Figure 3). The two strands form a short antiparallel β sheet with typical backbone hydrogen bonding (Figure 4A). In this specific region, the two protein chains cross over one another.

Recently, the nuclear magnetic resonance (NMR) structure of the soluble N-terminal conserved core of the murine cytomegalovirus (MCMV) homolog (M50) of pUL34 was reported (Leigh et al., 2015). As expected from the conservation analysis (Figure S2), the structures of MCMV M50 and pUL34 are highly similar, with a global root-mean-square deviation (RMSD) of ∼9 Å (Figures 4B and 4C). The most obvious difference between the two structures is the position of helix α4. In the unbound form (Leigh et al., 2015), helix α4 is in a closed conformation interacting with helix α1, whereas in the bound form, helix α4 is displaced into an open conformation, forming the groove for pUL31 helices α1 and α2. In essence, in the unbound state, pUL34 helix α4 occupies the position of pUL31 helix α2, while binding of pUL31 induces a major conformational change and displacement of pUL31 α4 and, concomitantly, a movement of helix α1. The hinge for this overall movement is the crossover region in Rɪɪɪ described earlier (Figure 4A). In the closed conformation, pUL34 strand β9 is shorter, and, upon binding, it extends and forms an antiparallel β sheet with pUL31 β1 (Figures 4A, 4D, and 4E).

In summary, in all three major interacting regions, the crucial residues are highly conserved, and mapping them onto the structure helps define their roles. The extensive analyses of site-specific mutants, using a range of different experimental systems, revealed that the interaction network between pUL31 and pUL34 is not redundant but rather complementary. This is evident because the individual mutation of most of the mentioned residues results in a loss of complex formation and, hence, function.

### Modeling of NEC Hexamer and In Situ Curved Hexagonal Lattice

**Figure 5:**
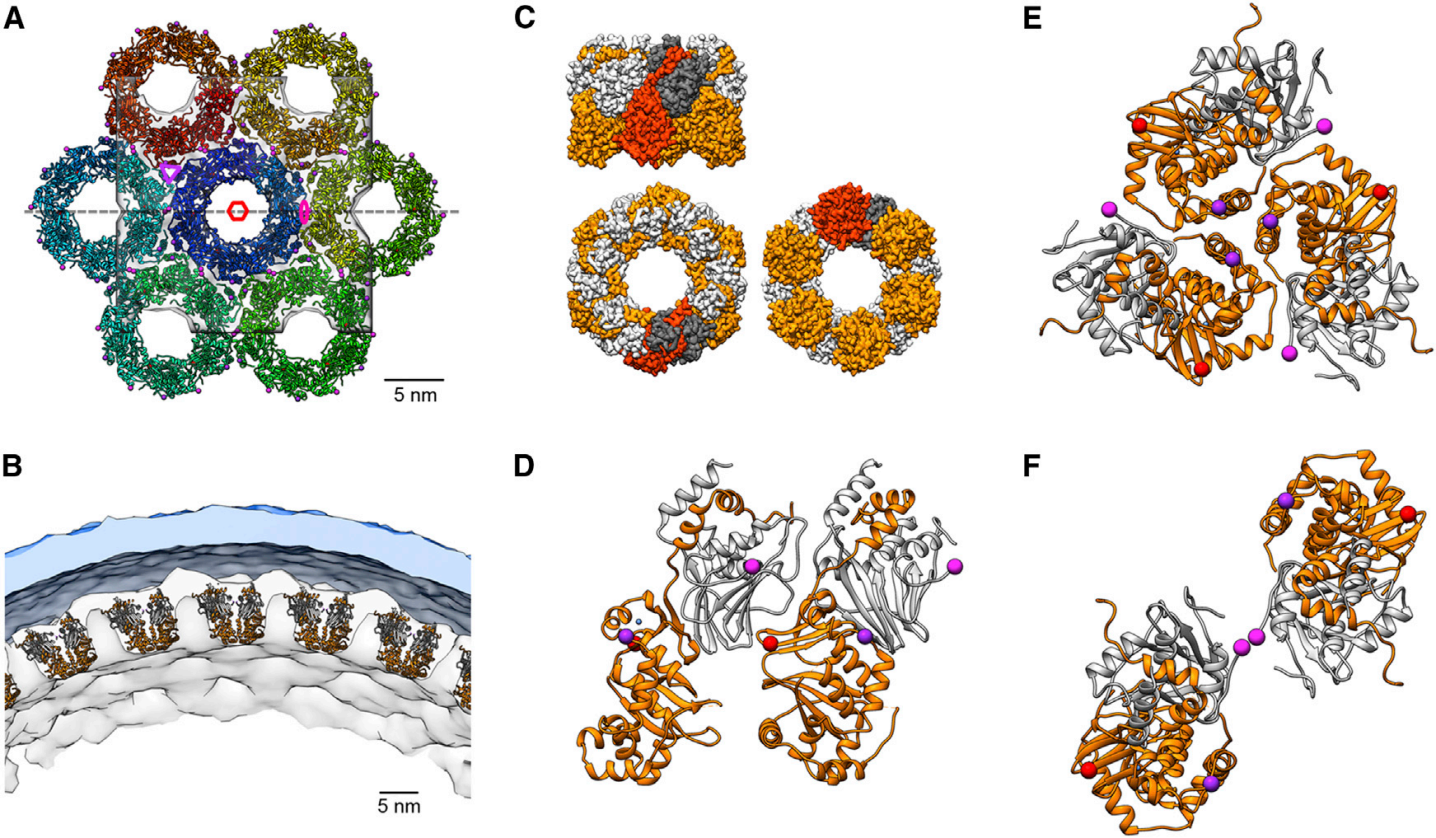
NEC Assembly to Curved Hexagonal Lattice The pUL31-pUL34 complex was fitted into the cryo-EM map obtained from visualizing the precisely curved NEC lattice in situ (Hagen et al., 2015). (A) Membrane-distal view (from vesicle interior) of the modeled hexameric lattice, consisting of 42 pUL31-pUL34 heterodimers arranged in 7 hexamers. The chains are rainbow colored. Dotted line depicts cutting planes used in (B). Red spheres indicate S77, magenta D22 and purple R101; cryoEM map in white. (B) “Side” view of the modeled lattice, rotated 90° relative to (A) and shown at a lower magnification to show a larger area of the lattice. The pUL31 chains are in orange; pUL34 is in gray. The inner-nuclear-membrane-derived vesicle membrane is shown on top (light blue). (C) A single hexamer of pUL31-pUL34 heterodimers. For one heterodimer, pUL31 is indicated in brick red, and pUL34 is indicated in dark gray, the remaining heterodimers are shown with pUL31 in orange and pUL34 in light gray. Top left: hexamer side view. Lower left: hexamer seen from the membrane. Lower right: hexamer seen from vesicle interior. (D–F) Close-up views on the inter-heterodimeric interfaces formed in the hexametric lattice. Colors are as in (A), and the light blue sphere indicates the Zn ion. (D) Side view of the dimer-dimer interface within the hexamer, indicated by the purple oval in (A). (E) Top view of trimer interface between hexamers (same view as in A; hexamer is indicated by magenta triangle in A). (F) Top view of inter-hexameric dimer interactions (same view as in A; hexamer is indicated by red hexagon in A).

The heterodimeric NEC self-assembles to form a coat with hexagonal characteristics to mediate capsid envelopment (Hagen et al. 2015). To study the assembly of the heterodimeric complex into a hexagonal lattice, the former was fitted into the cryo-EM map derived from cells forming native NEC-coated vesicles. A lattice model (Figure 5) was generated by global rotational search of the heterodimer and applying 6-fold symmetry; no further constraints were applied (for more details, see Experimental Procedures). The modeling resulted in the location of pUL34 most proximal to the membrane (Figures 5A–5C), thus validating the overall orientation of the fit. The long axis of the heterodimer is tilted by ∼70° relative to the membrane plane, i.e., being 20° off from a perpendicular orientation to the membrane (Figures 5C and 5D). This orientation further forms the basis for the interactions of the heterodimers in the hexamer. Our hexamer model indicates that pUL34 loop 88–93, as well as the pUL31 ZNF and loop 77–79, forms the intra-hexamer interface stabilized by hydrophobic interactions. This additional role for the ZNF motif is remarkable and underscores why it is so highly conserved. Support for this model comes from site-specific mutagenesis at pUL31 aa positions 76, 77, and 80 (Figure S4; Table S2). This also explains the unique phenotype of pUL31 S77, as this presents a highly conserved polar residue in an otherwise hydrophobic environment (Figure S2), and its effect on the pUL34 interaction (Table S2). Altogether, the pUL31 loop 77–79 can be considered as an important intra-hexamer interface.

To form a defined curved lattice that is key for successful budding as observed in situ (Hagen et al., 2015), the inter-hexamer interactions also have to be specific. These are established between hexamers at both trimer and dimer interfaces. Our model suggests that the hexamers’ trimeric interface is formed by pUL31 helix α5 and that the dimeric interface is formed by pUL34 loop 22–26, each interacting with their counterparts on the neighboring hexamer (Figures 5E and 5F). This is highly consistent with recent suggestions of residues in this region, for which a dominant-negative (DN) mutant D35A/E37A in HSV1 (Bjerke et al., 2003, Roller et al., 2010) was shown to impair curvature formation (Bigalke et al., 2014). The effect of the DN double mutant (corresponding numbers in PrV would be D22A/E24A) can be explained, since both the aspartic and glutamic acid residues are negatively charged and repulsive, thus promoting the lattice curvature. In the double mutant, these sites become non-polar and can undergo hydrophobic interactions, thus preventing curvature formation. While our global sequence alignment did not highlight pUL34 D22 and E24 as being particularly conserved, a local alignment would identify at least PrV E24 and HSV1 E37 as equivalent, as reported earlier (Roller et al., 2010).

Finally, the fit in lattice arrangement provides an indication where herpesvirus capsids as NEC cargo are likely to bind. The membrane-distal face of pUL31 is thereby suggested to form the inner coat of the cargo vesicles. Based on its electrostatic properties, cargo binding is likely to involve charged interactions (Movie S1, pUL31 acidic surface). Overall, the fit, based solely on a combined clash and protrusion score while not taking any mutation information as constraints, is consistent with previously reported data and results from our analyses presented here.

### Conclusions

pUL31 and pUL34 are conserved herpesviral proteins that form the highly stable heterodimeric NEC. During herpesvirus capsid nuclear egress, the heterodimers assemble into a hexagonal coat to mediate the formation of tight-fitting membrane vesicles around the capsids. The crystal structure of the complex presented here provides the molecular basis for both the heterodimer assembly and the formation of a curved hexagonal coat. Self-assembly of this coat at the inner nuclear membrane mediates capsid envelopment for transport across the nuclear envelope. In the future, the in situ cryo-EM data of NEC mutants stalled at the formation of a flat lattice, prior to the induction of curvature, will assist us in understanding the NEC changes required for membrane remodeling and vesicle formation. It will be interesting to identify alterations in the NEC that enable the curvature and the triggering event for this transition. From the extensive interactions within the heterodimer revealed here, it seems more likely that the relative angles between the rigid heterodimers, and between the heterodimers and the membrane, have to change for curvature formation, rather than the process being based on a hinge-like movement within the heterodimer. Finally, the structural analyses provided here give the blueprint of the necessary functional properties of cellular counterparts operational in the topologically related transport of large ribonucleoprotein particles (RNPs) through the nuclear envelope (Speese et al., 2012), for which the search is still ongoing.

## Experimental Procedures

### NEC Expression, Purification, and Crystallization

DNA coding for PrV pUL31 (UniProt: G3G955), lacking the N-terminal 25 aa (aa 26–271), and truncated PrV pUL34 (UniProt: G3G8X8; aa 1–179) with C-terminal His6 tag were cloned into a pETDuet plasmid. The NEC was expressed in *E. coli* strain LEMO21(DE3) cultivated in a medium containing 50 nM ZnCl at 25°C; expression was induced with 1 mM isopropyl β-D-1-thiogalactopyranoside (IPTG). Expression of selenomethionine-labeled NEC was as described earlier but with 5 nM ZnCl. NEC was purified by, first, heat treatment followed by affinity chromatography and size exclusion chromatography; for details, see the Supplemental Experimental Procedures. The complex crystallized in 15.5%–17% polyethylene glycol (PEG) 6000, 1 M lithium chloride, 0.1 M L-malic acid, MES, and Tris buffer (pH 6.2–7.2).

### Data Collection and Structure Determination

Diffraction data were recorded at the Diamond Light Source I04 beamline and processed with XIA2 (Kabsch, 2010). The structure was determined with the program PHENIX AUTOSOL (Adams et al., 2010), by single anomalous dispersion (SAD) phasing, using selenomethionine-labeled crystals. The model was built manually with the program COOT (Emsley and Cowtan, 2004) and restrained refinement (with TLS) with AUTOBUSTER (Bricogne, 2008). Final model geometry was checked with MolProbity (Davis et al., 2007). For data collection and refinement statistics, see Table S1.

### Generation and Characterization of pUL31 and pUL34 Mutants by In Situ Cellular Studies

Localization and co-localization of NEC mutants were performed as described by Klupp et al. (2007). Functional complementation was as recently described by Paßvogel et al. (2015). For a brief description, see the Supplemental Experimental Procedures.

### Fitting into EM Density

The NEC heterodimer was initially placed manually within the density of the sub-tomogram average (Hagen et al., 2015) constituting the central hexamer. A global rotational search (with a step size of ∼12° and a translational search of 4 Å in steps of 2 Å in the radial plane) was then performed to produce ∼140,000 different orientations for the heterodimer. Each reoriented heterodimer was symmetrized to produce a central hexamer; and, using a lattice spacing of 11.4 Å and a vesicle radius of 58 nm, six copies of the hexamer were modeled into the neighboring regions to produce a lattice. The presented model scored the highest using a combined score; for details, see the Supplemental Experimental Procedures.

## Author Contributions

T.Z., M.W., M.L., J.C., T.H., D.V., C.H., B.G.K., W.A., T.C.M., and K.G. intellectually conceived the experiments; T.Z., M.W., M.L., J.C., T.H., C.W., D.V., K.C.D., K.H., C.H., and B.G.K. performed the experiments; all authors processed and analyzed the data; and T.Z., C.H., W.A., T.C.M., and K.G. wrote the manuscript, and all authors commented on it.

## Accession Numbers

Atomic coordinates (PDB: 5E8C) as well as the hexagonal lattice model (PDB: 5FKI) were deposited to the PDB.

## Supplemental Information

Document S1. Supplemental Experimental Procedures, Figures S1–S5, and Tables S1 and S2

Movie S1. pUL31 Surface Electrostatics, Related to Figure 1

Movie S2. pUL34 Surface Electrostatics, Related to Figure 1

Document S2. Article plus Supplemental Information
