# Supplementary material for: Crystal Structure of the Herpesvirus Nuclear Egress Complex Provides Insights into Inner Nuclear Membrane Remodeling
Source: Cell Rep. 2015 Dec 17;13(12):2645–52. doi: 10.1016/j.celrep.2015.11.008 (PMC4700048; doi:10.1016/j.celrep.2015.11.008)
Supplement: Document S1. Supplemental Experimental Procedures, Figures S1–S5, and Tables S1 and S2 [file mmc1.pdf]

Cell Reports

Supplemental Information

Crystal Structure of the Herpesvirus Nuclear Egress Complex Provides Insights into Inner Nuclear Membrane Remodeling

Tzviya Zeev-Ben-Mordehai, Marion Weberruß, Michael Lorenz, Juliana Cheleski, Teresa Hellberg, Cathy Whittle, Kamel El Omari, Daven Vasishtan, Kyle C. Dent, Karl Harlos, Kati Franzke, Christoph Hagen, Barbara G. Klupp, Wolfram Antonin, Thomas C. Mettenleiter, and Kay Grünewald

SUPPLEMENTAL FIGURES

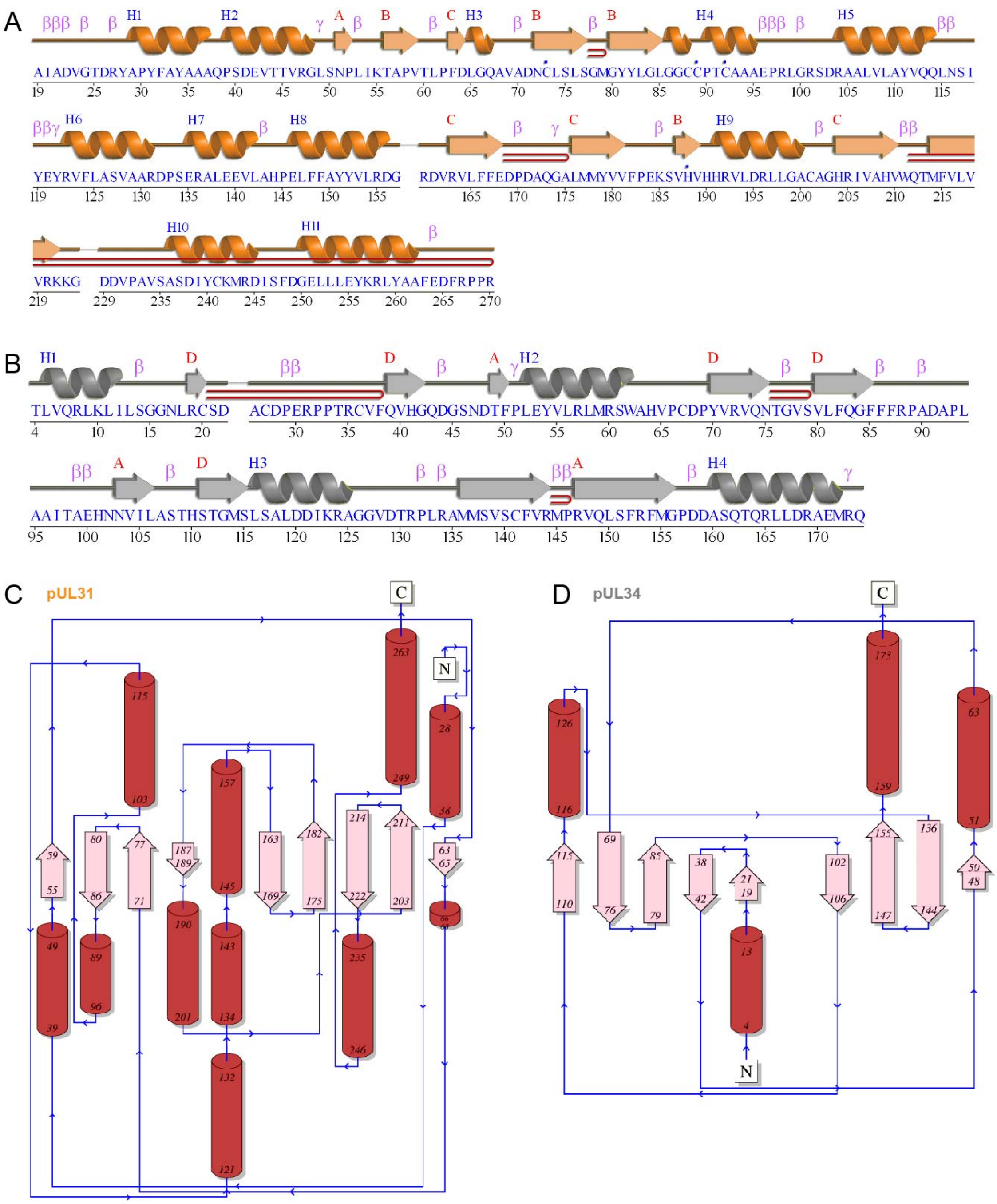

Figure S1. Secondary structure elements and topology mapping, related to Figure 1.
(A) and (B) Secondary structure elements mapped onto the sequence of pUL31 and pUL34, respectively. (C) and (D) topology maps of pUL31 and pUL34, respectively.

A

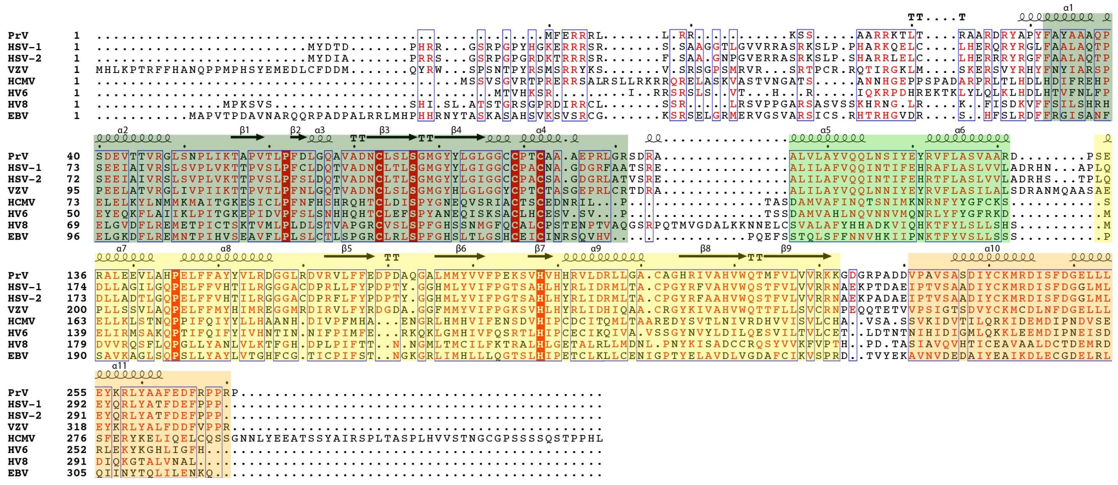

B

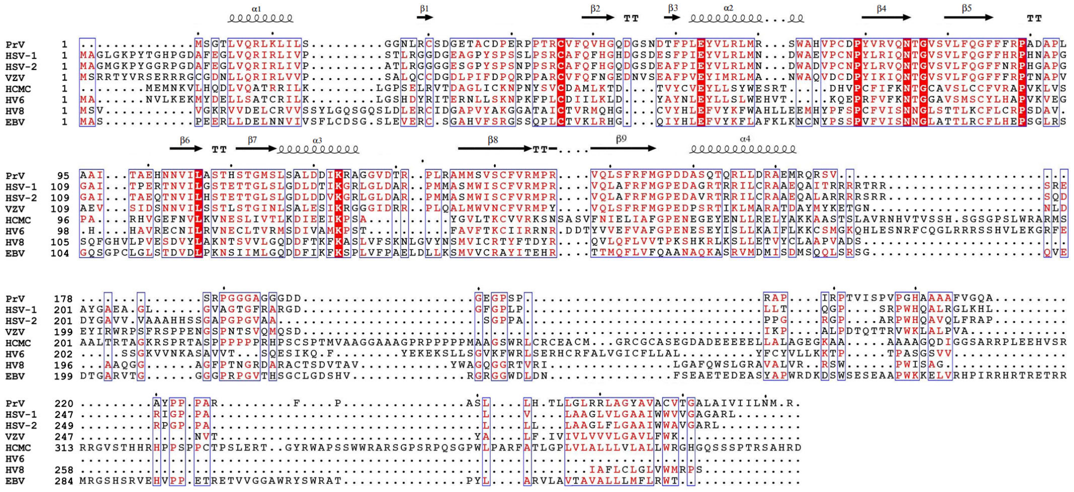

Figure S2. Multiple sequence alignment with selected homologs, related to Figure 1.

(A) Alignment for pUL31. The alignment includes pseudorabies virus (PrV; accession G3G955), human herpes simplex virus 1 (HSV1; accession P10215), human herpes simplex virus 2 (HSV2; accession P89454), human herpesvirus 3/ Varicella-zoster virus (VZV; accession P09283), human herpesvirus 5/ human cytomegalovirus (HCMV M53; accession F5HFZ4), human herpesvirus 6A/ human B lymphotropic virus (accession P28865), Human herpesvirus 8 type P (HV6; accession F5H982) and Epstein-Barr virus/ human herpesvirus 4 (HV4; accession P0CK48). Highly conserved residues are shaded and boxed. The four conserved regions CR1-CR4 1 (Lötzerich et al., 2006) are coloured: CR1, dark green; CR2, light green; CR3, yellow and CR4, orange.

(B) Alignment for pUL34. The alignment includes pseudorabies virus (PrV; accession G3G8GX), human herpes simplex virus 1 (HSV-1; accession P1021), human herpes simplex virus 2 (HSV-2; accession P89457), human herpesvirus 3/ Varicella-zoster virus (VZV; accession P09280), human herpesvirus 5/ human cytomegalovirus (HCMV M50; accession Q6SW81), human herpesvirus 6A/ human B lymphotropic virus (HV6; accession P52465), Human herpesvirus 8 type P (HV8; accession F5HA27) and Epstein-Barr virus/ human herpesvirus 4 (HV4; accession P03185).

The secondary structure elements of PrV virus are shown above the sequence, coloured and labelled per domain, every tenth amino acid is marked with a dot. Highly conserved residues are shaded and boxed. Clustal Omega (Sievers et al., 2011) and ESPript server (Robert and Gouet, 2014) were used for the alignment of representative herpesvirus homologs.

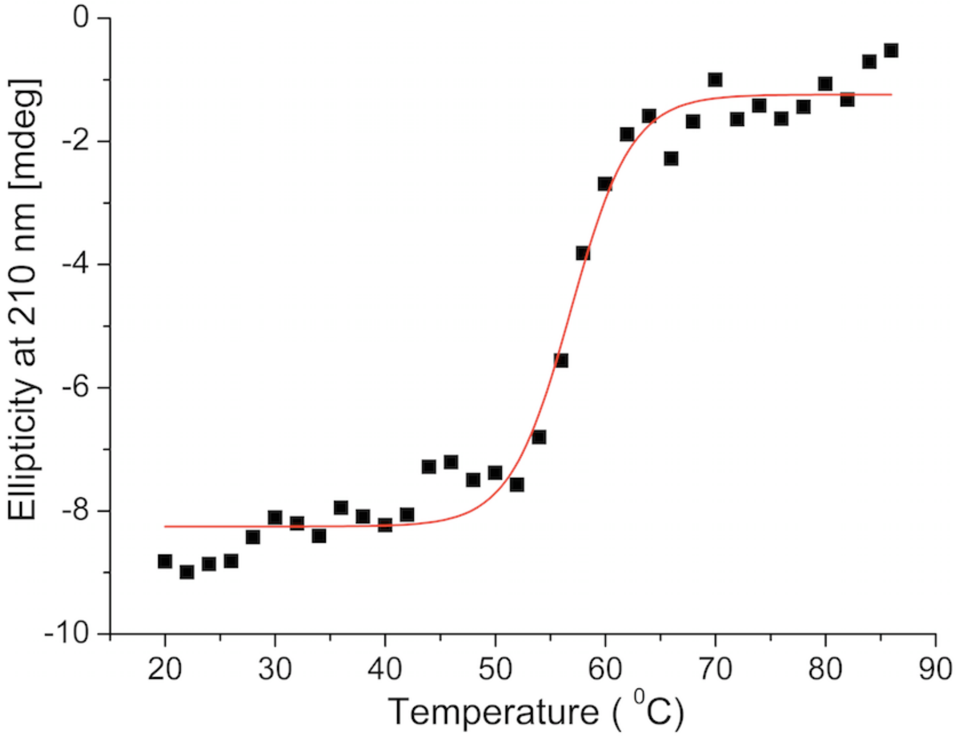

Figure S3. CD thermal denaturation profiles, related to Figure 3.
Thermal denaturation profiles of the NEC were recorded by circular dichroism. Melting temperature (Tm) is 57 °C. The denaturation is irreversible.

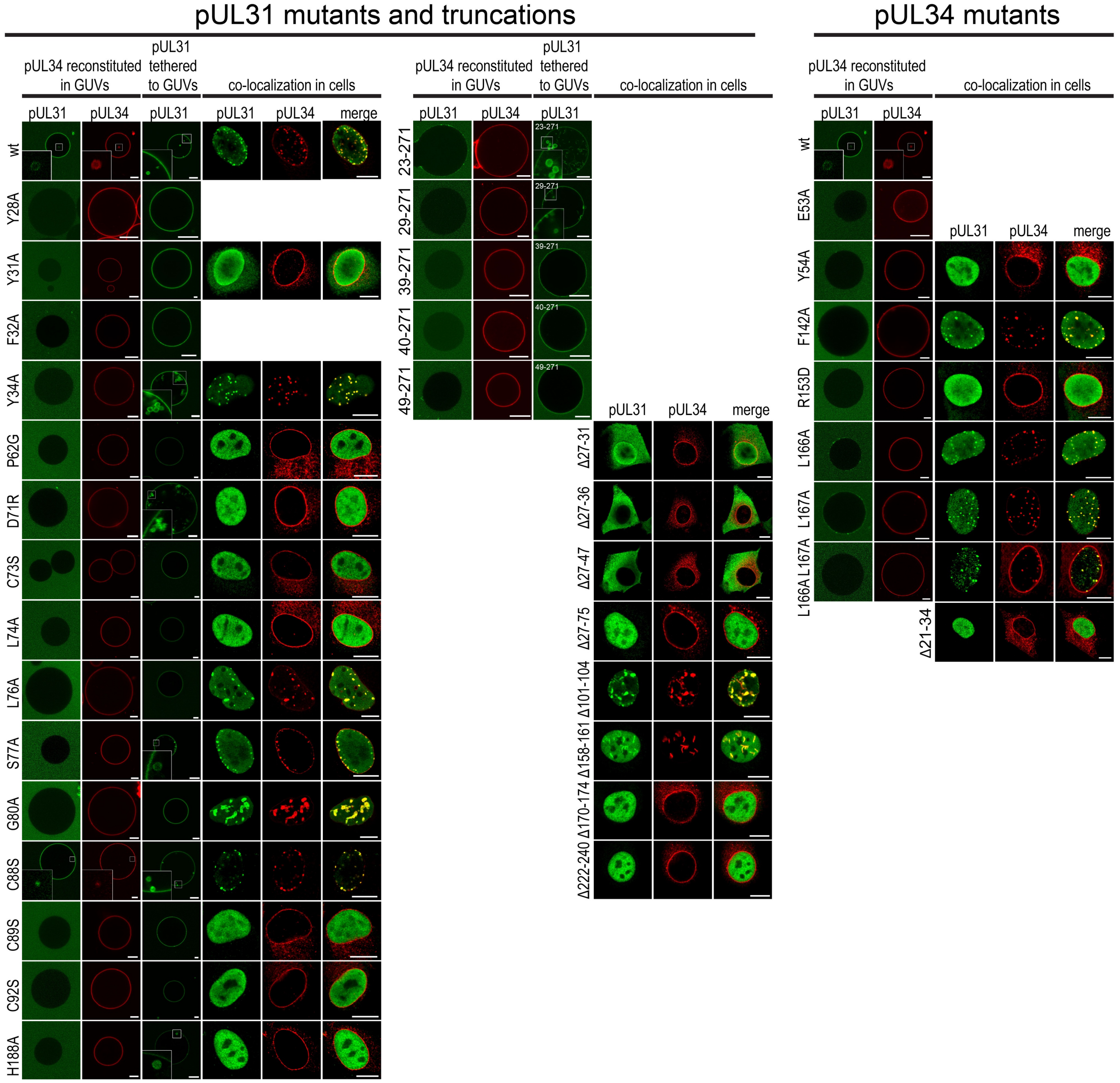

Figure S4. Summary of *in situ* and *in vitro* phenotypes of all reported mutations, related to Table S2, Figures 2 and 3.
pUL34 reconstituted in GUVs: Analysis of *in vitro* vesicle formation ability of pUL31 mutant on pUL34 giant unilamellar vesicles (GUVs). GUVs with reconstituted Alexa-546 labelled wildtype pUL34 (red channel) were incubated with EGFP-pUL31 wildtype or mutant proteins or EGFP (green channel). Binding of EGFP-pUL31 to pUL34 GUVs and intra-GUV vesicle formation were examined by confocal microscopy. Scale bars: 10 µm.
pUL31 tethered to GUVs: Analysis of *in vitro* vesicle formation ability of pUL31 mutants. His_6-EGFP, His_6-EGFP-pUL31 or His_6-EGFP-pUL31 mutants were directly tethered to GUVs containing Ni-NTA-DGS and vesicle formation was analysed by confocal microscopy. Scale bars: 10 µm.
Co-localization in cells: Rabbit kidney (RK13) cells were stably transfected with wildtype or mutant pUL31 encoding constructs (green channel) with wildtype pUL34 (red channel). Proteins were detected by immunofluorescence with respective antibodies followed by confocal microscopy. Scale bars: 10 µm.
Mutants are marked on the left of each raw.

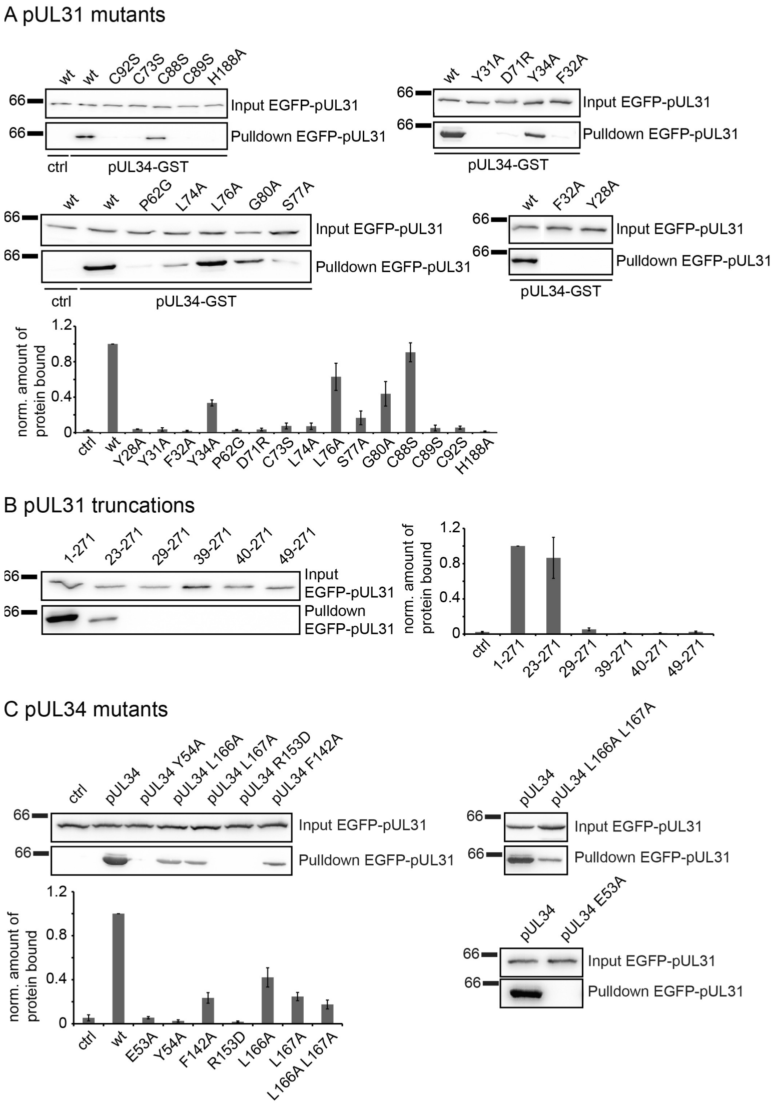

Figure S5. GST-pull-down assays for pUL31 and pUL34 mutants, related to Tables S2, Figures 2 and 3. Glutathion-S-Transferase (GST)-pull-downs of pUL31 mutants (A) and truncations (B). GST-pull-down experiments were performed using GST (ctrl) or GST-pUL34 (aa 1-240, TMR) as baits and EGFP-pUL31 *wt* and mutants as prey. GST-pUL34-bound EGFP-pUL31 was eluted with TEV protease containing buffer which cleaves the GST-fusions C-terminal of the GST tag. Input and eluate were analysed by western blotting using anti-EGFP-antibodies. Pull-downs of three independent experiments were quantified and the amount of eluted mutant pUL31 is shown normalized to *wt* pUL31. Error bars: SEM (C) GST-pull-downs of pUL34 mutants. GST-pull-down experiments were performed using GST (ctrl), GST-pUL34 (aa 1-240, TMR) or the respective mutants as baits. *wt* EGFP-pUL31 served as prey. GST-pUL34-bound EGFP-pUL31 was eluted with TEV protease containing buffer. Input and eluate were analysed by Western blotting using anti-EGFP-antibodies. Pull-downs of three independent experiments were quantified and the amount of eluted *wt* pUL31 bound to pUL34 versions is shown normalized to *wt* pUL34. Error bars: SEM

Table S1. Table of data collection and structure refinement statistics, related to Figure 1

**Data collection**	
Beamline	DLS-I04
Space group	*P* 4_3 2 2
Unit cell parameters (Å,°)	a=b=91.69, c=108.26<br>α=β=γ=90
No. of crystals	5
Wavelength	0.9790
Resolution (Å)	70.0-2.9 (2.98-2.90)
No. of unique reflections	10771 (777)
Completeness (%)	99.9 (99.9)
Multiplicity	227.3 (209.1)
Anomalous completeness	99.9 (99.9)
Anomalous multiplicity	125.3 (111.6)
I/σ(I)	35.6 (4.2)
R_{merge} (%)	30.8 (346.6)
R_{pim} (%)	2.0 (23.8)
CC half	1.0 (0.93)
**Refinement**	
Resolution (Å)	50.0-2.9
R_{work}/R_{free} (%)	21.9/26.5
R.m.s.d. bond (Å)	0.007
R.m.s.d. angle (º)	0.90
Mean B-factors/Wilson plot (Å^2)	92/87
Number of atoms	3175
Metal (Zn)	1
Total number of residues	406
Ramachandran plot (%)<br>favoured/allowed/outliers	95.7/100/0

Table S2. **Phenotypes of mutants analyzed, related to Figure 2 and Figure S4**

Position	Vesicle formation on pUL34 GUV*	Vesicle formation if directly tethered**	pUL31 binding to pUL34 in GST-pulldowns	Localization in transfected cells	Colocalization pUL31-pUL34 in co-transfected cells	Complementa-tion of PrV-DUL31	Electron microscopy analysis of complementation assays
**in pUL31**							
wt	pos.	pos.	100%	nucleus	pos.	pos.	all stages of virion maturation (Fuchs et al., 2002)
Y28A	neg.	neg.	0%				
Y31A	neg.	strongly reduced	0%	nucleus	neg.	neg.	
F32A	neg.	neg.	0%				
Y34A	neg.	pos.	30%	nucleus	pos.	reduced	
P62G	neg.	neg.	0%	nucleus	neg.	neg.	Capsids dispersed in nucleus
D71R	neg.	pos.	0%	nucleus	neg.	neg.	
C73S	neg.	neg.	10%	nucleus	neg.	neg.	Capsids dispersed in nucleus
L74A	neg.	neg.	10%	nucleus	neg.	neg.	Capsids dispersed in nucleus
L76A	neg.	neg.	60%	nucleus	pos.	neg.	Large infoldings of NM; capsids at INM; no budding
S77A	neg.	reduced	20%	nucleus	pos.	neg.	Capsids at INM; no budding
G80A	neg.	neg.	40%	nucleus	pos.	neg.	Large infoldings of NM; capsids at INM; no budding
C88S	pos.	pos.	90%	nucleus	pos.	pos.	all stages of virion maturation
C89S	neg.	neg.	10%	nucleus	neg.	neg.	Capsids dispersed in nucleus
C92S	neg.	neg.	10%	nucleus	neg.	neg.	Capsids dispersed in nucleus
H188A	neg.	reduced	0%	nucleus	neg.	neg.	Capsids dispersed in nucleus
aa23-271	reduced	reduced	90%				
aa29-271	neg.	reduced	10%				
aa39-271	neg.	strongly reduced	0%				
aa40-271	neg.	neg.	0%				
aa49-271	neg.	neg.	0%				
CR1 Δ5 (Δ27-31)				nucleus	neg.	neg.	

CR1 Δ10 (Δ27-36)				nucleus + cytoplasm	neg.	neg.	
CR1 Δ20 (Δ27-47)				nucleus + cytoplasm	neg.	neg.	
CR1 Δ50 (Δ27-75)				nucleus + cytoplasm	neg.	neg.	
Δ101-104				nucleus	pos.	neg.	
Δ158-161				nucleus	pos.	neg.	
Δ170-174				nucleus	neg.	neg.	
Δ222-240				nucleus	neg.	neg.	
**in pUL34**							
wt	pos.		100%	rim	pos.	pos.	all stages of virion maturation (Klupp et al., 2000)
E53A	neg.		10%				
Y54A	neg.		0%	rim	neg.	neg.	
E53AY54A				rim	neg.	neg.	Capsids dispersed in nucleus
F142A	neg.		20%	rim	pos.	reduced	
R153D	neg.		0%	rim	neg.	neg.	
L166A	reduced		40%	rim	pos.	pos.	
L167A	reduced		20%	rim	pos.	reduced	
L166AL167A	neg.		20%	rim	pos.	neg.	Capsids dispersed in nucleus
Δ2-4 (N5)				rim	pos.	pos.	
Δ2-9 (N10)				rim	neg.	neg.	
Δ21-34				rim + ER	neg.	(unstable)	

* induction of vesicles by pUL31 on GUVs with reconstituted pUL34 (pos.: pUL31 binding to the GUV membranes and induction of intra-GUV vesicles identical to wildtype level; reduced: only weak pUL31 binding to pUL34-GUVs, intra-GUV vesicles rare; neg.: no pUL31 binding to pUL34-GUVs, no vesicle induction).

** Induction of intra-GUV vesicles if pUL31 is directly tethered to the GUV membrane (pos.: number of vesicle containing GUVs 80-100% of wildtype level; reduced: number of vesicle containing GUVs 40-80% of wildtype level; strongly reduced: number of vesicle containing GUVs 10-40% of wildtype level; negative: no vesicle induction)

SUPPLEMENTAL MOVIE LEGENDS

Movie S1. pUL31 surface electrostatics, related to Figure 1.

Movie S2. pUL34 surface electrostatics, related to Figure 1.

SUPPLEMENTAL EXPERIMENTAL PROCEDURES

Purification and Crystallization

NEC was purified by first heat treatment (42 °C for 15 minutes) followed by clarification (25,000*g*, 15 min, 16 °C). Next the complex was purified by affinity chromatography (Co^{2+}-Sepharose 6-fast flow) and size exclusion chromatography in 10 mM Tris-HCl pH7.4, 75 mM NaCl, 3 mM DTT and 0.1mM TCEP. The complex was concentrated to ~15 mg/ml and 5 mM TCEP-HCL was added before crystallisation. Crystals were flash frozen using 25 % (v/v) glycerol/reservoir solution as cryo-protectant.

Circular dichroism spectroscopy

Circular dichroism (CD) measurements were performed on a JASCO J-815 CD spectropolarimeter (JASCO Corporation, Hachioji-shi, Tokyo, Japan). Denaturation of NEC were monitored at 210 nm in the temperature range 20–85 °C with a concentration of 0.1 mg/ml, scanning speed of 50 nm/min, 10 mM phosphate buffer, 20 mM NaCl, pH 7.4. Data analysis was carried out using Origin 7.0. The thermal denaturation data were fit to a derivation of the Boltzmann equation for the two-state unfolding model using Origin 7.0 to obtain the midpoint of denaturation (the thermal melting point).

GUV *in vitro* mutant characterisation

Details are described in (Lorenz et al., 2015). In brief, constructs for expression of PrV pUL31 and pUL34 were generated from a synthetic DNA optimized for codon usage in *E. coli* (Geneart) and pUL31 mutants were generated on this basis using the Quick change mutagenesis kit (Agilent). pUL31 and pUL34 proteins were expressed and purified, GUVs generated and GST pull-down assays analysed as described (Lorenz et al., 2015).
Alexa Fluor 546 carboxylic acid succinimidyl ester was obtained from Life Technologies and detergents from Calbiochem. The nuclear envelope lipid mix consists of 5 mol % cholesterol, 2.5 mol % sphingomyeline, 2.5 mol % Na-phosphatidylserine, 10 mol % Na-phosphatidylinositol, 20 mol % phosphatidylethanolamine, and 60 mol % phosphatidylcholine (all from Avanti Polar Lipids) .

Generation and characterization of pUL31 and pUL34 mutants by cellular studies *in situ*

Site directed mutagenesis was used to generate single amino acid substitutions as well as internal deletion mutants by modifying either pcDNA-UL31 (Fuchs et al., 2002) or pcDNA-UL34 (Klupp et al., 2000) using the QuickChange II XL site-directed mutagenesis kit (Agilent Technologies) as described recently (Paßvogel et al., 2013).
To test for localization of the mutated proteins rabbit kidney (RK13) cells were transfected with the corresponding expression plasmids by calcium phosphate co-precipitation (Graham and van der Eb, 1973). Transfected cells were fixed with 3 % paraformaldehyde / 0.3 % TritonX100 two days after transfection and labelled either with the polyclonal rabbit anti-pUL31 (Fuchs et al., 2002) or the polyclonal rabbit anti-pUL34 serum (Klupp et al., 2000), which were diluted 1:500 in PBS. After incubation at room temperature for 1 h, bound antibody was detected with Alexa Fluor 488 goat anti-rabbit antibodies (Invitrogen).
For co-localization studies, RK13 cells were co-transfected with the mutated plasmids and the corresponding plasmid expressing the wild-type complex partner. To simultaneously detect pUL31 and pUL34, transfected cells were fixed as described above and incubated with rabbit polyclonal anti-pUL31 and mouse polyclonal anti-pUL34 antisera (Klupp et al., 2007) Alexa Fluor 488 anti-rabbit and Alexa Fluor 555 anti-mouse antibodies (Invitrogen) were applied to detect bound anti-pUL31 and anti-pUL34 antibodies, respectively. All secondary antibodies were diluted 1:1,000 in PBS. Fluorescence images were recorded with a laser scanning confocal microscope (SP5; Leica, Mannheim, Germany).
Cell lines stably expressing mutant pUL31 or pUL34 were generated after transfection of RK13 cells with the corresponding expression plasmids. Two days post transfection cells were split and incubated with G418 (0.5 µg/µl) containing medium to select for transfected cells. After 12 to 14 days cell colonies were visible and picked by aspiration. Single cell clones were tested for pUL31 or pUL34 expression by indirect immunofluorescence and by western blotting using the corresponding monospecific rabbit sera as described above. Cell clones efficiently expressing the mutated proteins were propagated and used for single step growth kinetics.
To test for functional complementation RK13 cells expressing wild-type or mutated pUL31 or pUL34 were infected with PrV-*wt* (PrV strain Kaplan; Kaplan and Vatter (1959)) and the corresponding deletion mutants PrV-ΔUL31 (Fuchs et al., 2002) or PrV-ΔUL34 (Klupp et al., 2000) with a multiplicity of infection of 5. 24h post infection cells and supernatant were harvested and titrated on RK13-UL31 or RK13-UL34 cells, respectively. One-step growth assays were repeated three times and mean values were calculated. Results were ranked as follows: Titres comparable to PrV-*wt* from the corresponding cell line or at maximum 10-fold reduced as positive, titres reduced between 10- and 50-fold as reduced, titres lower than 50-fold but more than 10-fold than those from non-complementing cells as strongly reduced, and titres equalling those of the respective deletion mutant on RK13 cells (negative control) as negative.
To investigate the defect in greater detail, mutant pUL31 or pUL34 expressing cells were infected with the corresponding deletion mutant at a multiplicity of infection of 1. Cells were fixed and processed for transmission electron microscopy 14 hours post infection as described previously (Klupp et al., 2000).

Scores used for the fitting into EM density

To determine which model best fit the map, two scores were used. First was a ‘protrusion score’ that measured the percentage of backbone atoms of a single heterodimer that were placed in voxels with intensity less than 2 (*i.e.* sitting outside the envelope of the map). Second was a clash score, which measured the percentage of all atoms of a single heterodimer that were within 1.2 Å of an atom from a neighbouring heterodimer. These scores were summed and the models ranked accordingly. The model shown here had the lowest combined protrusion and clash score.
