# Supplementary figures and images for: Crystal Structure of the Herpesvirus Nuclear Egress Complex Provides Insights into Inner Nuclear Membrane Remodeling
Source: Cell Rep. 2015 Dec 17;13(12):2645–52. doi: 10.1016/j.celrep.2015.11.008 (PMC4700048; doi:10.1016/j.celrep.2015.11.008)

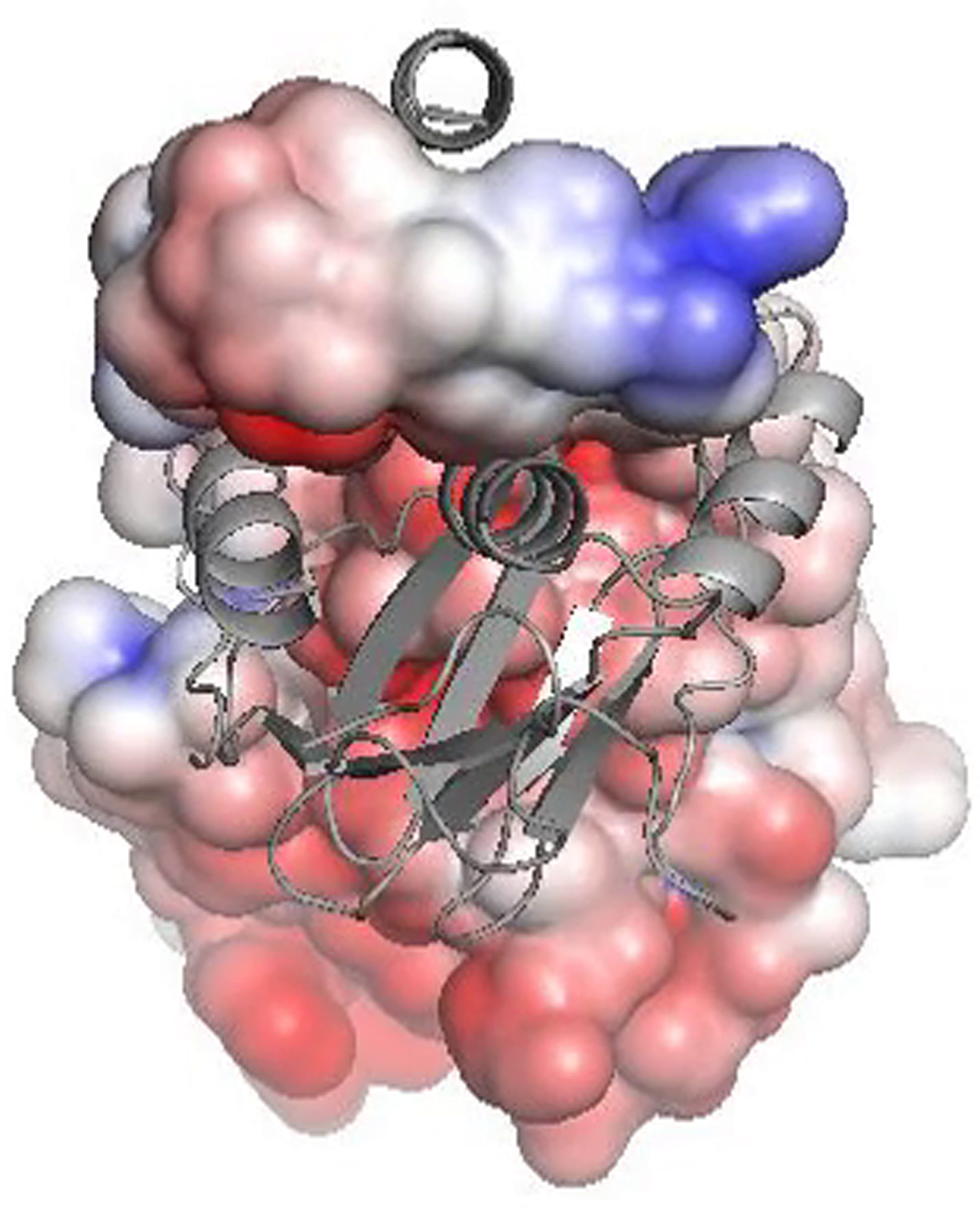

Supplement: Movie S1. pUL31 Surface Electrostatics, Related to Figure 1 [file mmc2.jpg]

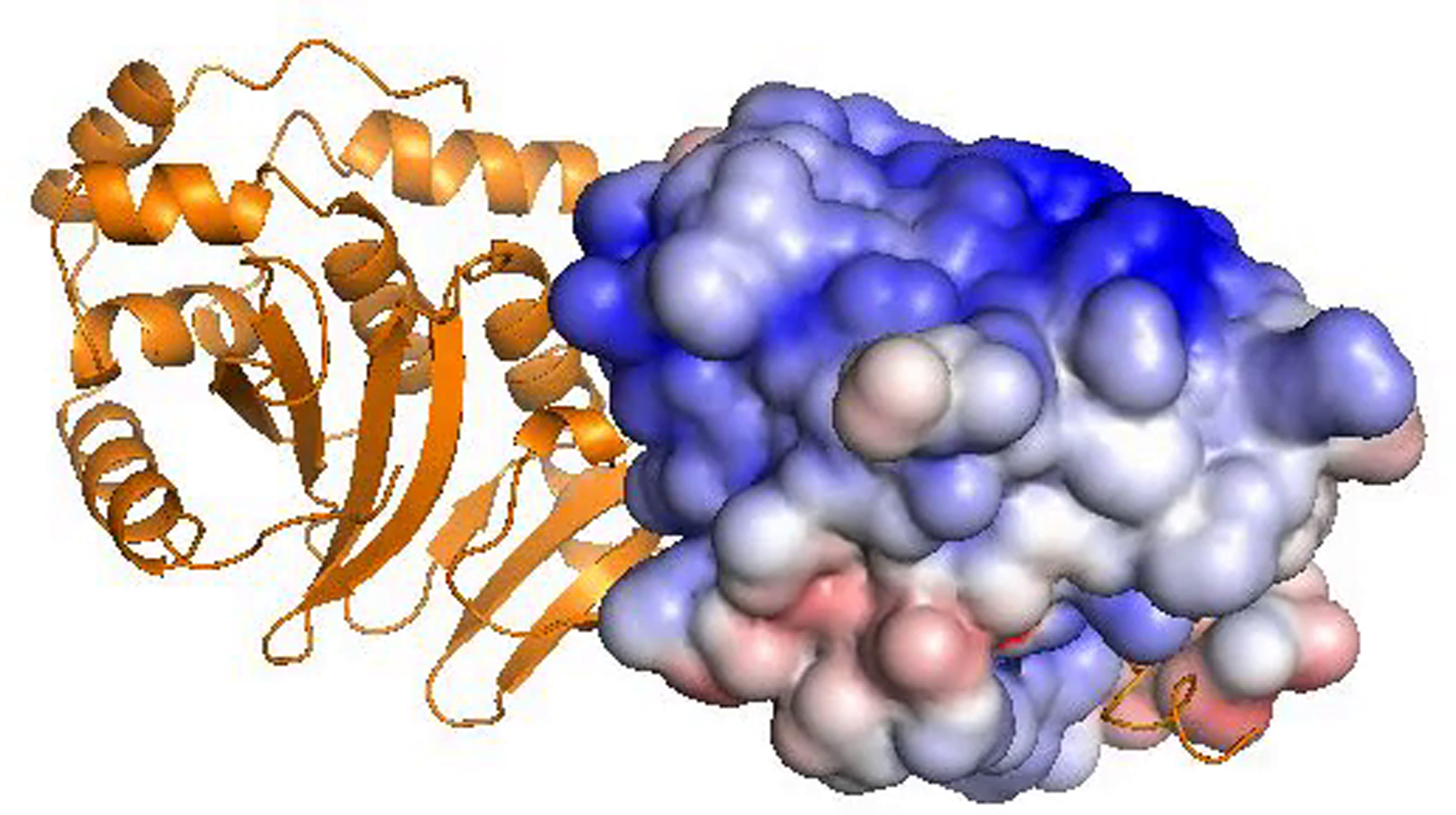

Supplement: Movie S2. pUL34 Surface Electrostatics, Related to Figure 1 [file mmc3.jpg]
