# Supplementary material for: Crystal Structure of the Herpesvirus Nuclear Egress Complex Provides Insights into Inner Nuclear Membrane Remodeling
Source: Cell Rep. 2015 Dec 17;13(12):2645–52. doi: 10.1016/j.celrep.2015.11.008 (PMC4700048; doi:10.1016/j.celrep.2015.11.008)
Supplement: Document S2. Article plus Supplemental Information [file mmc4.pdf]

Cell Reports

Report

Crystal Structure of the Herpesvirus Nuclear Egress Complex Provides Insights into Inner Nuclear Membrane Remodeling

Graphical Abstract

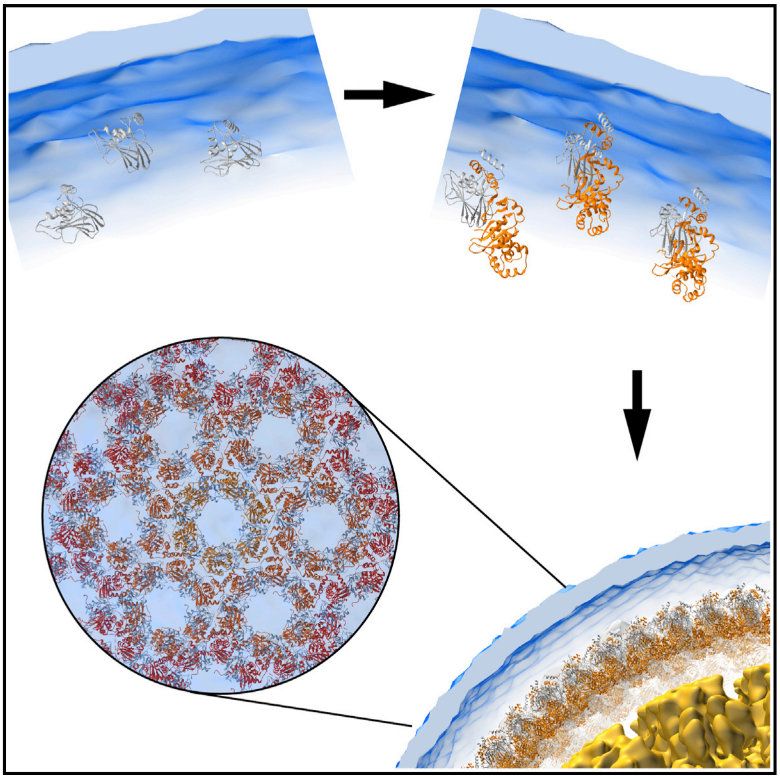

Highlights

- Both components of the nuclear egress complex, pUL31 and pUL34, have a unique fold
- pUL31 has a conserved C3H zinc finger motif
- The nuclear egress complex is highly stable with an extensive interaction surface
- Molecular basis of interactions to assemble a curved hexagonal lattice coat is revealed

Authors

Tzviya Zeev-Ben-Mordehai, Marion Weberruß, Michael Lorenz, ..., Wolfram Antonin, Thomas C. Mettenleiter, Kay Grünewald

Correspondence

wolfram.antonin@tuebingen.mpg.de (W.A.),
kay@strubi.ox.ac.uk (K.G.)

In Brief

Herpesvirus capsids exit the nucleus by a vesicular transport through the nuclear double membrane. Zeev-Ben-Mordehai et al. present the crystal structure of the coat proteins mediating envelopment of capsids. The structure is used to model the assembly of hexagonal lattice driving vesicle formation at the inner nuclear membrane.

Accession Numbers

5E8C
5FKI

Zeev-Ben-Mordehai et al., 2015, Cell Reports *13*, 2645–2652
December 29, 2015 ©2015 The Authors
http://dx.doi.org/10.1016/j.celrep.2015.11.008

CellPress

Cell Reports
Report

OPEN ACCESS
CellPress

Crystal Structure of the Herpesvirus Nuclear Egress Complex Provides Insights into Inner Nuclear Membrane Remodeling

Tzviya Zeev-Ben-Mordehai,[1,4] Marion Weberruß,[2,4] Michael Lorenz,[2,4] Juliana Cheleski,[1,4] Teresa Hellberg,[3,4] Cathy Whittle,[1] Kamel El Omari,[1] Daven Vasishtan,[1] Kyle C. Dent,[1] Karl Harlos,[1] Kati Franzke,[3] Christoph Hagen,[1] Barbara G. Klupp,[3] Wolfram Antonin,[2,*] Thomas C. Mettenleiter,[3] and Kay Grünewald[1,*]
[1]Division of Structural Biology, Wellcome Trust Centre for Human Genetics, University of Oxford, Roosevelt Drive, Oxford OX3 7BN, UK
[2]Friedrich Miescher Laboratory of the Max Planck Society, 72076 Tübingen, Germany
[3]Institute of Molecular Virology and Cell Biology, Friedrich-Loeffler-Institut, Federal Research Institute for Animal Health, 17493 Greifswald – Insel Riems, Germany
[4]Co-first author
*Correspondence: wolfram.antonin@tuebingen.mpg.de (W.A.), kay@strubi.ox.ac.uk (K.G.)
http://dx.doi.org/10.1016/j.celrep.2015.11.008
This is an open access article under the CC BY license (http://creativecommons.org/licenses/by/4.0/).

SUMMARY

Although nucleo-cytoplasmic transport is typically mediated through nuclear pore complexes, herpesvirus capsids exit the nucleus via a unique vesicular pathway. Together, the conserved herpesvirus proteins pUL31 and pUL34 form the heterodimeric nuclear egress complex (NEC), which, in turn, mediates the formation of tight-fitting membrane vesicles around capsids at the inner nuclear membrane. Here, we present the crystal structure of the pseudorabies virus NEC. The structure revealed that a zinc finger motif in pUL31 and an extensive interaction network between the two proteins stabilize the complex. Comprehensive mutational analyses, characterized both in situ and in vitro, indicated that the interaction network is not redundant but rather complementary. Fitting of the NEC crystal structure into the recently determined cryoEM-derived hexagonal lattice, formed in situ by pUL31 and pUL34, provided details on the molecular basis of NEC coat formation and inner nuclear membrane remodeling.

INTRODUCTION

Viruses have developed remarkable strategies to usurp cellular processes for their own needs while maintaining efficient virus production. This is particularly obvious in the life cycle of herpesviruses, which replicate viral DNA and assemble nucleocapsids in the nucleus of non-dividing cells. These capsids are translocated from the nucleoplasm to the cytoplasm via vesicular trafficking through the nuclear envelope, thereby bypassing nuclear pore complexes, the canonical gateways of nucleo-cytoplasmic transport. The translocation of capsids across the nuclear double membrane is a two-step process; first, capsids are enveloped at the inner nuclear membrane, a process referred to as primary envelopment. These enveloped capsids are then fused with the outer nuclear membrane and thereby released into the cytoplasm (for reviews, see Johnson and Baines, 2011; Mettenleiter et al., 2013).

Envelopment of herpesvirus capsids requires extensive restructuring of the host inner nuclear membrane to form capsid-containing vesicles. Budding and scission of membrane vesicles in intracellular trafficking are meditated by dedicated coat proteins, such as clathrin and the COP I and COP II complexes, which induce membrane curvature on the cytosolic membrane face to ultimately extrude cytoplasmic vesicles (McMahon and Gallop, 2005). Vesicle formation in the opposite direction, i.e., away from the cytosol, is less prevalent but does occur. Examples include the invagination of the endosomal membrane during the formation of multi-vesicular bodies and the egress of HIV and other enveloped viruses at the plasma membrane, as mediated by the endosomal sorting complexes required for transport (ESCRT) machinery (Votteler and Sundquist, 2013). Herpesvirus vesicle formation into the nuclear envelope lumen belongs to this latter category, because it is directed away from the nucleoplasm, which is topologically equivalent to the cytoplasm.

It is well accepted that herpesvirus capsid nuclear egress is mediated by two conserved herpesvirus proteins, designated as pUL31 and pUL34, in the alphaherpesviruses herpes simplex virus 1 (HSV1) and pseudorabies virus (PrV) (Johnson and Baines, 2011; Mettenleiter et al., 2013). pUL34 is a type II single-pass transmembrane protein residing in the endoplasmic reticulum and nuclear membranes, while pUL31 is a soluble protein targeted to the nucleoplasm by a nuclear localization signal. In the nucleus of infected cells, pUL34 recruits pUL31 to the inner nuclear membrane to form the nuclear egress complex (NEC). Both proteins are required for the transport of viral capsids through the nuclear envelope and, thus, for productive herpesvirus replication (Bubeck et al., 2004; Fuchs et al., 2002; Klupp et al., 2000; Reynolds et al., 2001). Although the participation of other viral and cellular proteins in perinuclear vesicle formation during infection cannot be excluded, transient or stable co-expression of both proteins in eukaryotic cells results in the generation of periplasmic vesicles originating from the inner nuclear membrane, even in the absence of capsids

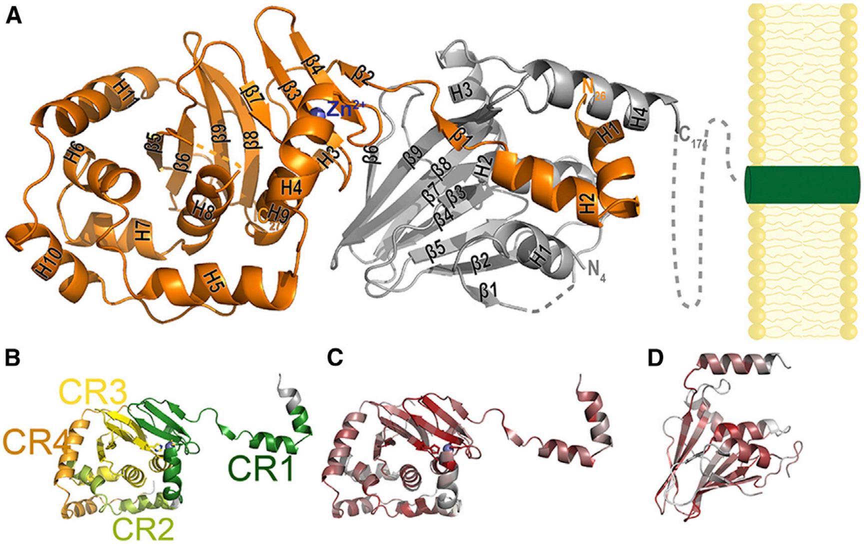

Figure 1. Structure and Conservation of the NEC

(A) The pUL31-pUL34 complex in cartoon representation. pUL31 is shown in orange, and pUL34 is shown in gray. Unresolved regions are indicated by dashed lines, and the pUL31 bound Zn^{2+} ion is indicated in blue. Secondary structure elements are labeled on the structure (see also Figure S2). The inner nuclear membrane (yellow) and the transmembrane region of pUL34 (green) are schematically drawn.

(B) pUL31 structure colored according to the previously described conserved regions CR1–CR4 (Lötzerich et al., 2006). CR1, dark green; CR2, light green; CR3, yellow; CR4, orange. The Zn^{2+} ion and its complexing residues, shown in stick representation, are indicated.

(C) Mapping of the sequence conservation for pUL31 (see sequence alignment in Figure S2A), ranging from dark red for the most conserved to light gray for the least conserved residues.

(D) Mapping of the sequence conservation for pUL34 (see sequence alignment in Figure S2B). Color code is as in (C).

See also Figure S2.

(Klupp et al., 2007). Moreover, pUL31 and pUL34 are sufficient for budding of small vesicles and scission into giant unilamellar vesicles (GUVs) (Bigalke et al., 2014; Lorenz et al., 2015). Furthermore, in this minimal model membrane system, artificial tethering of pUL31 to GUV membranes induced its self-oligomerization, which resulted in vesicle budding and scission into the GUV lumen (Lorenz et al., 2015). Thus, the NEC represents self-sufficient machinery that can mediate inwardly directed vesicle formation.

Here, we present the crystal structure of the PrV NEC, revealing that both components have a unique fold and that pUL31 contains a zinc finger motif. The structure, with its extensive interaction surface, explained the complex's high stability. The crystal structure was further fitted into the recently determined cryoelectron microscopy (cryo-EM) hexagonal lattice formed in situ by pUL31 and pUL34 (Hagen et al., 2015), providing details on the intra- and inter-hexamer interactions leading to formation of the NEC coat.

RESULTS AND DISCUSSION

Structure Determination

To produce high-quality diffracting crystals of the PrV NEC, a pUL31 construct (amino acids [aa] 26–271) lacking the nuclear localization signal was co-expressed in *E. coli*, together with the conserved region of pUL34 (aa 1–179). Data were collected for a selenomethionine derivative of the complex, the electron density map was calculated from experimental phasing, and the structure was refined to a 2.9 Å resolution (see Table S1 for the statistics for diffraction data collection and refinement of the atomic model). One heterodimer was present in the asymmetric unit. For pUL31, the complete aa chain was traced, with the exception of disordered loops at positions 158–159, 173–174, and 224–228 and residue 101. For pUL34, the chain was traced from aa 4 to 174, with the exception of a disordered loop at positions 23–25. The complex structure is overall ordered, with helices α5 and α10 of pUL31 being the most mobile parts of the complex.

Both pUL31 and pUL34 Have a Unique Fold

The crystal structure of the heterodimer displays an elongated shape of ~50 Å in width and ~100 Å in length, with the putative membrane-distal end being composed of pUL31 and the membrane-proximal part being composed mainly of pUL34 (Figure 1). This shape is highly similar to that of the heterodimer determined in solution by small-angle X-ray scattering (Hagen et al., 2015). Each protein of the heterodimer is, overall, globular, with the exception of helices α1 and α2 of pUL31, which extend around pUL34. The topology of both proteins seems to be unique (Figure S1), and no structural homologs could be found in the protein database (based on Dali server searches against the PDB).

pUL34 has a β sandwich fold comprising nine β strands. Strands β3, β6, β8, and β9 form one sheet of the fold that faces pUL31, and strands β1, β2, β4, β5, and β7 form the other sheet. Four helices cap the fold on the membrane-proximal side. Both the N and the C termini of pUL34 are located on the membrane-proximal side of the molecule (Figure 1A).

pUL31 has a complex topology (Figure 1; Figure S1), with 11 helices and nine β strands. Its core is made of helices α6–α8 and strands β5–β9 and is surrounded by four helices (α4–α5 and α10–α11). Coloring the protein according to the previously defined conserved regions (CR) 1 to 4 (Lötzerich et al., 2006) confirmed that CR1 is mainly involved in interaction with pUL34, while CR3 represents the structural core (Figure 1B). Notably, a C3H zinc finger (ZNF) motif is present, with three cysteines contributed by CR1 and a histidine by CR3. Mapping of conserved aa onto the structure revealed conservation of further sites and structural elements for each protein (Figures 1C and 1D; Figure S2). A subsequent detailed analysis of the structure highlighted the nature of these specific sites and motives.

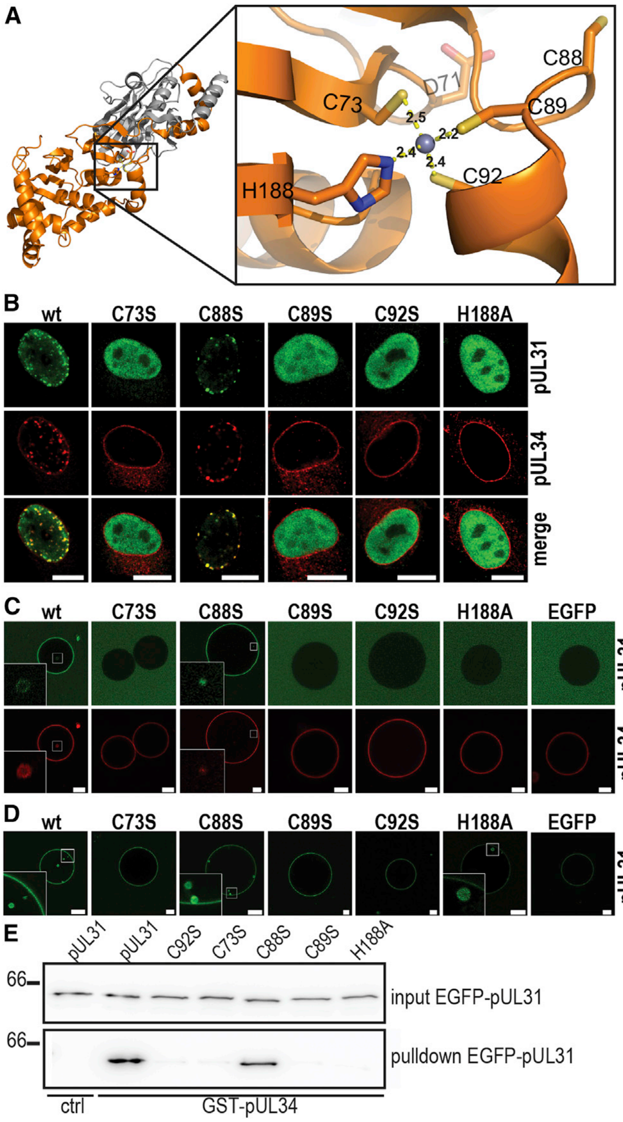

Figure 2. pUL31 ZNF Motif Is Vital for NEC Function

(A) Close-up view on the pUL31 ZNF motif. The side chains Cys73, Cys89, Cys92, and His188 coordinating the Zn^{2+} ion are shown as sticks, and respective distances (in angstroms) are indicated. Colors are as described in Figure 1A.

(B) In situ phenotype of pUL31 mutants. RK13 cells were co-transfected with WT pUL34 and pUL31 WT or mutant constructs. Proteins were detected by immunofluorescence with respective antibodies followed by confocal microscopy of the nuclear region. Anti-pUL31 is shown in green; anti-pUL34 is shown in red. Scale bars, 10 μm.

(C and D) In vitro phenotype of mutations in the ZNF motif affecting vesicle formation. (C) Soluble WT pUL31 (expressed as an EGFP fusion) was added to pUL34-GUVs. Recruitment of pUL31 and vesicle formation into the GUV lumen was assayed. Green channel shows pUL31-EGFP; red channel shows Alexa Fluor 546-labeled pUL34. (D) His_6-tagged-EGFP or His_6-tagged-EGFP-pUL31 proteins were directly tethered to Ni-NTA-DGS containing GUVs, in the absence of pUL34. Scale bars, 10 μm.

(E) Western blot of pull-downs showing pUL31-pUL34 interactions. Anti-EGFP antibodies were used to detect pull-downs using GST (ctrl) or GST-pUL34 as baits and EGFP-pUL31 WT and mutants as prey (for details, see Experimental Procedures).

pUL31 ZNF Motif Is Crucial for the Heterodimer Formation and Function

The four residues coordinating the Zn^{2+} ion (C73, C89, C92, and H188) belong to the most conserved residues in pUL31 (Figure 1C; Figure S2). ZNFs are important compact structural motifs that are known to function as interaction modules (Kochańczyk et al., 2015). The ZNF motif in pUL31 is on the surface facing pUL34 (Figure 2A). To define the function of the ZNF motif in pUL31, cysteines C73, C89, and C92 were individually replaced by serines, and the histidine H188 was replaced by an alanine, and functional analyses of the mutant proteins were performed both in vitro with artificial membranes and in situ in eukaryotic cells. In cells co-expressing the two proteins, wild-type (*wt*) pUL31 is recruited to the inner nuclear membrane by the membrane-anchored pUL34, and characteristic "speckles" were observed. pUL31 mutants defective in binding of pUL34 have been shown to mis-localize diffusely to the nucleoplasm (Klupp et al., 2007). To assess the impact of the ZNF residues on the function of pUL31, each of the ZNF mutants, as well as a serine substitution of the adjacent (C88S), was co-expressed with wild-type (WT) pUL34 in rabbit kidney (RK13) cells. While the pUL31 C88S mutant co-localized with pUL34 at the nuclear rim and showed characteristic WT-like "speckles," all ZNF mutants exhibited diffuse nucleoplasmic localization (Figure 2B; Table S2), indicating a defect in interaction.

To further define whether the ZNF mutants directly affect the membrane deformation activity of pUL31, we used the recently established in vitro system in which full-length pUL34 is reconstituted into artificial membranes; i.e., GUVs (Lorenz et al., 2015). Addition of soluble WT pUL31 (expressed as an EGFP-fusion protein) to GUVs containing pUL34 (pUL34-GUVs) resulted in the recruitment of the protein to the GUV surface and induced vesicle budding and scission into the GUV lumen, a process that is topologically identical to the NEC-mediated vesicle formation at the nuclear envelope (Figure 2C). In contrast to WT pUL31, all four ZNF mutants failed to bind pUL34-GUV membranes or induce vesicle formation. Consistent with the results from the cellular localization assay, the C88S mutant did not affect binding to pUL34-GUVs and induced WT pUL31-like formation of intra-GUV vesicles.

The failure of pUL31 mutants to induce vesicle formation in pUL34 GUVs could be caused by defects in their membrane remodeling activity and/or in their ability to bind pUL34, possibly as a result of severe misfolding of the protein. To distinguish between the two possibilities, the ability of pUL31 mutants to bind pUL34 was analyzed by a GST (glutathione S-transferase)-pull-down assay, using pUL34 lacking the transmembrane region as bait. All four pUL31 ZNF mutants failed to bind pUL34, while the C88S mutant behaved like WT (Figure 2E; Table S2). This is in agreement with the cellular localization assay and the pUL34-GUV in vitro data and confirms that lack of interaction caused the observed phenotypes.

To test whether the ZNF residues not only affect the interaction with pUL34 but also the membrane remodeling activity of pUL31, WT pUL31 and the mutant proteins were each directly tethered to GUV membranes, bypassing the need for pUL34 for membrane anchorage (for details, see Supplemental Experimental Procedures and Lorenz et al., 2015). Recruitment of WT pUL31

OPEN
ACCESS
CellPress

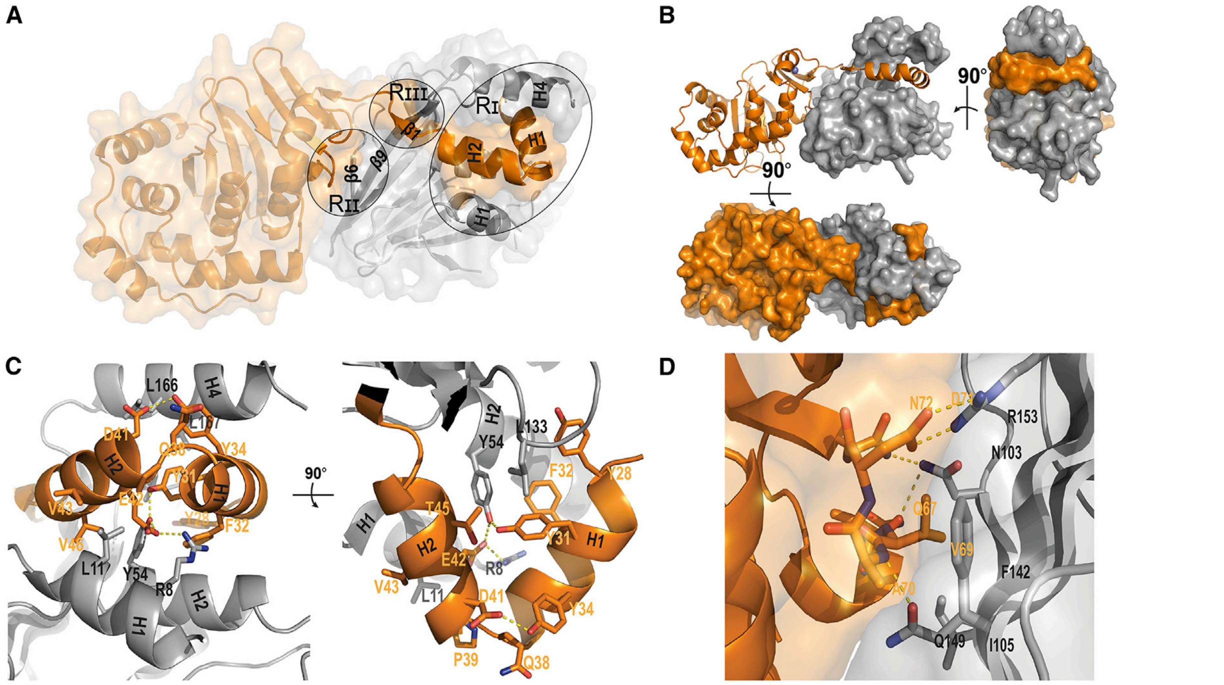

Figure 3. The NEC Has an Extensive Interaction Surface that Can Be Divided into Three Regions

(A) Overview of the three interaction regions, marked RI–RIII. Colors are as in Figure 1A.

(B) Three orthogonal views of the complex highlighting the extent of the pUL31 N-terminal "arm."

(C and D) Close-up views on regions I and II, respectively. Residues involved in the interaction are shown in sticks; hydrogen bonds are indicated by yellow dotted lines.

and the C88S mutant resulted in intra-GUV vesicle formation. In contrast, proteins mutated in C73, C89, or C92 were defective in membrane remodeling. Interestingly, the H188A mutant was able to induce vesicle formation, but to a lower extent than WT (Figure 2D; Table S2).

PrV pUL31 mutants defective in nuclear envelope vesicle formation do not support herpesvirus replication (Paßvogel et al., 2015). Indeed, only WT pUL31 and mutant pUL31 C88S showed full complementation, i.e., viral titers similar to WT PrV in a trans-complementation assay of pUL31-deleted PrV on RK13 cells stably expressing the mutated proteins (Table S2). In contrast, all four pUL31 ZNF mutants could not functionally substitute for WT pUL31. Taken together, these results indicate that the ZNF motif is indispensable for NEC formation and the membrane remodeling necessary for the budding and scission of vesicles.

The NEC Is a Very Stable Complex with an Extensive Interaction Surface

All attempts to dissociate the purified complex with high salt and mild detergents were unsuccessful (data not shown). While high-level expression of a pUL34 (aa 1–179) fragment by itself was successful, pUL31 could not be efficiently overexpressed alone, suggesting that pUL31 is not stable in the absence of pUL34. In contrast, the heterodimer proved to be very stable, as measured by circular dichroism, giving a high melting temperature of 57°C (Figure S3). The extensive buried surface area on both proteins (~1,850 Å^2, corresponding to ~13%, for pUL31; and ~1,750 Å^2, corresponding to 18%, for pUL34) is likely the cause of the observed high stability of the complex. The two proteins have specific intermolecular hydrogen bonds in three regions that are further enforced by hydrophobic interactions (Figure 3). These three regions were analyzed in more detail.

Region I: Extended pUL31 Angular "Arm" Fitting into a pUL34 Groove

The most striking interacting region, region I (RI), is that of a long N-terminal pUL31 angular "arm" formed by helices $\alpha1$ and $\alpha2$, that fits tightly into a specific groove in pUL34 (Figures 1 and 3B). The ~80° angle between the helices that make the arm reach around pUL34 in a "hugging" fashion is stabilized by hydrogen bonds between the side chains of Y34 and D41 (Figure 3B). The interaction of pUL31 with pUL34 in this region is mainly restricted to three residues on pUL34 helices $\alpha1$ and $\alpha2$, viz., pUL34 R8 and L11 on helix $\alpha1$ and Y54 on helix $\alpha2$. pUL34 Y54 is key for this interaction region, forming hydrogen bonds with both pUL31 Y31 and E42. Interaction with pUL34 helix $\alpha4$, forming the other side of the groove, is mainly hydrophobic and mediated through L167. A hydrophobic pocket is formed by residues from both pUL34 and pUL31, particularly pUL34 L133 and pUL31 F32 (Figure 3C). The importance of these residues for the interaction was verified by site-specific mutagenesis. Mutations in pUL31 at position Y31 abolished pUL34 binding, whereas mutating position Y34 strongly reduced the interaction as evident in GST pull-down assays (Figure S5; Table S2). Consistently, both of these pUL31 mutants failed to bind to pUL34-GUVs (Figure S4; Table S2). As indicated, Y54 of

pUL34 is key for the interaction in Rɪ, and, accordingly, mutating this residue abolished pUL31 binding (Figure S4; Table S2). Mutating L167 to an alanine strongly reduced pUL31 binding. Both pUL34 mutants also failed to recruit WT pUL31 either completely (pUL34 Y54A) or recruited it less efficiently (L167A) when reconstituted into GUVs (Figure S4; Table S2). Accordingly, pUL31 Y31A was not recruited to the nuclear envelope by WT pUL34 in co-transfected cells, and pUL34 Y54A failed to recruit WT pUL31 (Figure S4; Table S2). Both mutant proteins were also unable to complement the absence of the respective WT protein in virus-infected cells (Table S2). The pUL31 Y34A and pUL34 L167A mutations showed, in this respect, a less severe phenotype. In agreement with its reduced but not abolished capability, pUL31 Y34A was still recruited to the nuclear envelope in the presence of pUL34. Similarly, pUL34 L167A can recruit pUL31 to the nuclear envelope, and both proteins can replace the corresponding WT protein in complementation assays, although less efficiently.

Notably, all the residues involved in Rɪ are highly conserved (Figures 1C and 1D; Figure S2), consistent with their crucial function in the interaction of both proteins. The very tight fit of the pUL31 arm into the pUL34 groove ensures a unique specificity and avidity for the interaction between the NEC partners (Movies S1 and S2).

Region ɪɪ: Interaction Surface in the Core of the Complex

Region ɪɪ (Rɪɪ) is formed by the pUL31 loop connecting helix $\alpha3$ and $\beta3$ comprising residues 67–72 and pUL34 residues from $\beta6$ and $\beta9$ (Figure 3D). pUL31 residue D71 forms a strong salt bridge with two hydrogen bonds to pUL34 R153 in pUL34 $\beta9$ (Figure 3D). N103 from pUL34 $\beta6$ has side chain and backbone interactions with pUL31 N72 and Q67, respectively. This region also exhibits hydrophobic interactions between pUL34 V69 and F142 from pUL34 $\beta9$ and I105 from $\beta6$.

The importance of the D71-R153 salt bridge for heterodimer formation was validated by site-specific mutagenesis. pUL31 D71 was substituted to an arginine (D71R), and pUL34 R153 was mutated to aspartic acid (R153D) and assayed by GST pull-down. Mutation of either of these residues abolished the interaction with the respective WT counterpart (Figure S4; Table S2). Consistent with this, pUL31 D71R was not recruited to pUL34-GUVs and failed to induce vesicle formation (Figure S4; Table S2). Similarly, WT pUL31 did not bind to GUVs that displayed reconstituted pUL34 R153D (Figure S4; Table S2). Interestingly, pUL31 D71R was able to induce vesicle formation if directly tethered to GUVs, indicating that the mutation does not affect the pUL31 membrane remodeling activity but specifically inhibits pUL34 binding (Figure S4; Table S2). Consistent with these results, pUL31 D71R was not recruited to the nuclear envelope by WT pUL34 in co-transfected cells. pUL34 R153D, despite showing a nuclear envelope staining, could not recruit WT pUL31 to the nuclear envelope and was also unable to complement the defect of the deletion mutant (Figure S4; Table S2). Mutating pUL34 phenylalanine at position 142 to alanine (F142A) resulted in a severe interaction phenotype, but not to the same extent as mutating the D71-R153 salt bridge. pUL34 F142A reconstituted into GUVs failed to recruit WT pUL31; hence, no vesicle formation was detectable (Figure S4; Table S2). In the GST pull-down assay, binding of pUL31 was reduced to about 20% of WT levels (Figure S5; Table S2). Whereas pUL34 F142A localized to the nuclear rim of transfected cells, the recruitment of WT pUL31 was impaired but not abolished, which is consistent with the remaining interaction level observed in GST-pull-downs (Figure S5; Table S2). Together, the interactions in Rɪɪ form a central interaction surface. Notably, pUL31 residue D71 is in very close proximity to the ZNF motif, particularly C73 (Figure 2A); thus, the structural integrity ensured by the ZNF motif directly affects the interactions in Rɪɪ.

Region ɪɪɪ: Crossover between pUL31 and pUL34 Chains

A third interacting region (Rɪɪɪ) is formed by interaction of the end of the very long pUL34 $\beta9$ strand (aa 154–157) with the short pUL31 $\beta1$ strand (Figure 3). The two strands form a short antiparallel β sheet with typical backbone hydrogen bonding (Figure 4A). In this specific region, the two protein chains cross over one another.

Recently, the nuclear magnetic resonance (NMR) structure of the soluble N-terminal conserved core of the murine cytomegalovirus (MCMV) homolog (M50) of pUL34 was reported (Leigh et al., 2015). As expected from the conservation analysis (Figure S2), the structures of MCMV M50 and pUL34 are highly similar, with a global root-mean-square deviation (RMSD) of $\sim$9 Å (Figures 4B and 4C). The most obvious difference between the two structures is the position of helix $\alpha4$. In the unbound form (Leigh et al., 2015), helix $\alpha4$ is in a closed conformation interacting with helix $\alpha1$, whereas in the bound form, helix $\alpha4$ is displaced into an open conformation, forming the groove for pUL31 helices $\alpha1$ and $\alpha2$. In essence, in the unbound state, pUL34 helix $\alpha4$ occupies the position of pUL31 helix $\alpha2$, while binding of pUL31 induces a major conformational change and displacement of pUL31 $\alpha4$ and, concomitantly, a movement of helix $\alpha1$. The hinge for this overall movement is the crossover region in Rɪɪɪ described earlier (Figure 4A). In the closed conformation, pUL34 strand $\beta9$ is shorter, and, upon binding, it extends and forms an antiparallel β sheet with pUL31 $\beta1$ (Figures 4A, 4D, and 4E).

In summary, in all three major interacting regions, the crucial residues are highly conserved, and mapping them onto the structure helps define their roles. The extensive analyses of site-specific mutants, using a range of different experimental systems, revealed that the interaction network between pUL31 and pUL34 is not redundant but rather complementary. This is evident because the individual mutation of most of the mentioned residues results in a loss of complex formation and, hence, function.

Modeling of NEC Hexamer and In Situ Curved Hexagonal Lattice

The heterodimeric NEC self-assembles to form a coat with hexagonal characteristics to mediate capsid envelopment (Hagen et al. 2015). To study the assembly of the heterodimeric complex into a hexagonal lattice, the former was fitted into the cryo-EM map derived from cells forming native NEC-coated vesicles. A lattice model (Figure 5) was generated by global rotational search of the heterodimer and applying 6-fold symmetry; no further constraints were applied (for more details, see Experimental Procedures). The modeling resulted in the location of

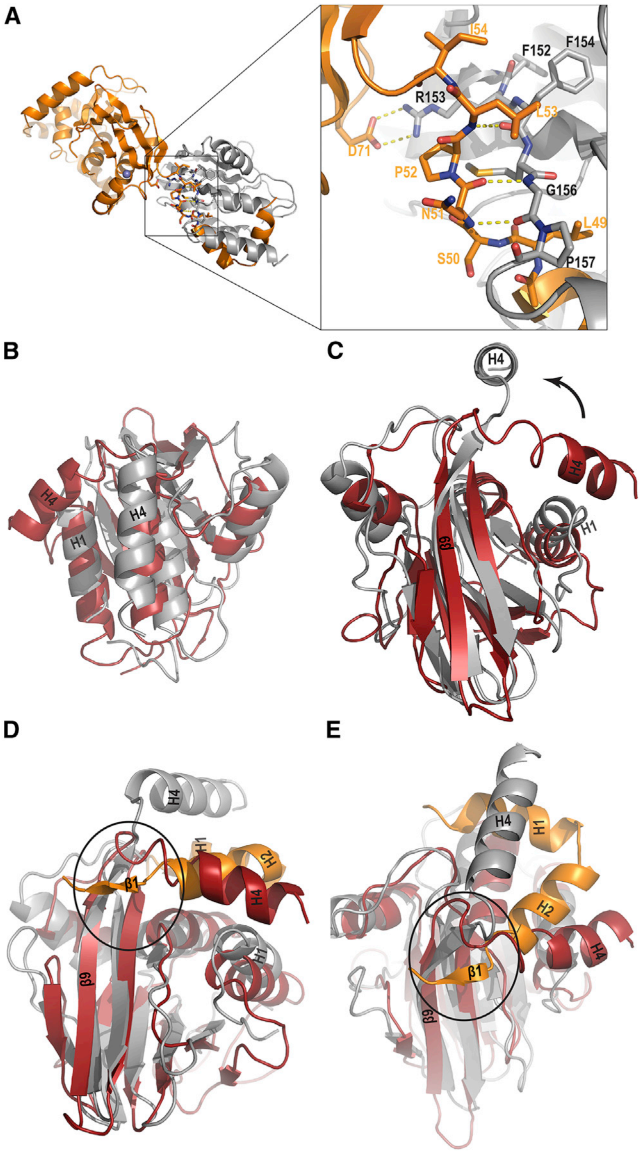

Figure 4. Binding of pUL31 Induces a Major Conformational Change in pUL34

(A) Residues involved in RIII are shown in sticks, and hydrogen bonds are indicated by dotted lines. Colors as in Figure 1A.

(B–E) Superposition of the mouse CMV pUL34 homolog M50 (PDB: 5A3G) on the complex. (B and C) Two orthogonal views of the superposition showing M50 in red and pUL34 within the NEC structure in gray. (D and E) Two views of the superposition that include pUL31 helix α1, α2, and β1 in orange. Ovals indicate region RIII.

pUL34 most proximal to the membrane (Figures 5A–5C), thus validating the overall orientation of the fit. The long axis of the heterodimer is tilted by ~70° relative to the membrane plane, i.e., being 20° off from a perpendicular orientation to the membrane (Figures 5C and 5D). This orientation further forms the basis for the interactions of the heterodimers in the hexamer. Our hexamer model indicates that pUL34 loop 88–93, as well as the pUL31 ZNF and loop 77–79, forms the intra-hexamer interface stabilized by hydrophobic interactions. This additional role for the ZNF motif is remarkable and underscores why it is so highly conserved. Support for this model comes from site-specific mutagenesis at pUL31 aa positions 76, 77, and 80 (Figure S4; Table S2). This also explains the unique phenotype of pUL31 S77, as this presents a highly conserved polar residue in an otherwise hydrophobic environment (Figure S2), and its effect on the pUL34 interaction (Table S2). Altogether, the pUL31 loop 77–79 can be considered as an important intra-hexamer interface.

To form a defined curved lattice that is key for successful budding as observed in situ (Hagen et al., 2015), the inter-hexamer interactions also have to be specific. These are established between hexamers at both trimer and dimer interfaces. Our model suggests that the hexamers' trimeric interface is formed by pUL31 helix α5 and that the dimeric interface is formed by pUL34 loop 22–26, each interacting with their counterparts on the neighboring hexamer (Figures 5E and 5F). This is highly consistent with recent suggestions of residues in this region, for which a dominant-negative (DN) mutant D35A/E37A in HSV1 (Bjerke et al., 2003; Roller et al., 2010) was shown to impair curvature formation (Bigalke et al., 2014). The effect of the DN double mutant (corresponding numbers in PrV would be D22A/E24A) can be explained, since both the aspartic and glutamic acid residues are negatively charged and repulsive, thus promoting the lattice curvature. In the double mutant, these sites become non-polar and can undergo hydrophobic interactions, thus preventing curvature formation. While our global sequence alignment did not highlight pUL34 D22 and E24 as being particularly conserved, a local alignment would identify at least PrV E24 and HSV1 E37 as equivalent, as reported earlier (Roller et al., 2010).

Finally, the fit in lattice arrangement provides an indication where herpesvirus capsids as NEC cargo are likely to bind. The membrane-distal face of pUL31 is thereby suggested to form the inner coat of the cargo vesicles. Based on its electrostatic properties, cargo binding is likely to involve charged interactions (Movie S1, pUL31 acidic surface). Overall, the fit, based solely on a combined clash and protrusion score while not taking any mutation information as constraints, is consistent with previously reported data and results from our analyses presented here.

Conclusions

pUL31 and pUL34 are conserved herpesviral proteins that form the highly stable heterodimeric NEC. During herpesvirus capsid nuclear egress, the heterodimers assemble into a hexagonal coat to mediate the formation of tight-fitting membrane vesicles around the capsids. The crystal structure of the complex presented here provides the molecular basis for both the heterodimer assembly and the formation of a curved hexagonal coat. Self-assembly of this coat at the inner nuclear membrane mediates capsid envelopment for transport across the nuclear envelope. In the future, the in situ cryo-EM data of NEC mutants stalled at the formation of a flat lattice, prior to the induction of curvature, will assist us in understanding the NEC changes required for membrane remodeling and vesicle formation. It will

OPEN ACCESS
CellPress

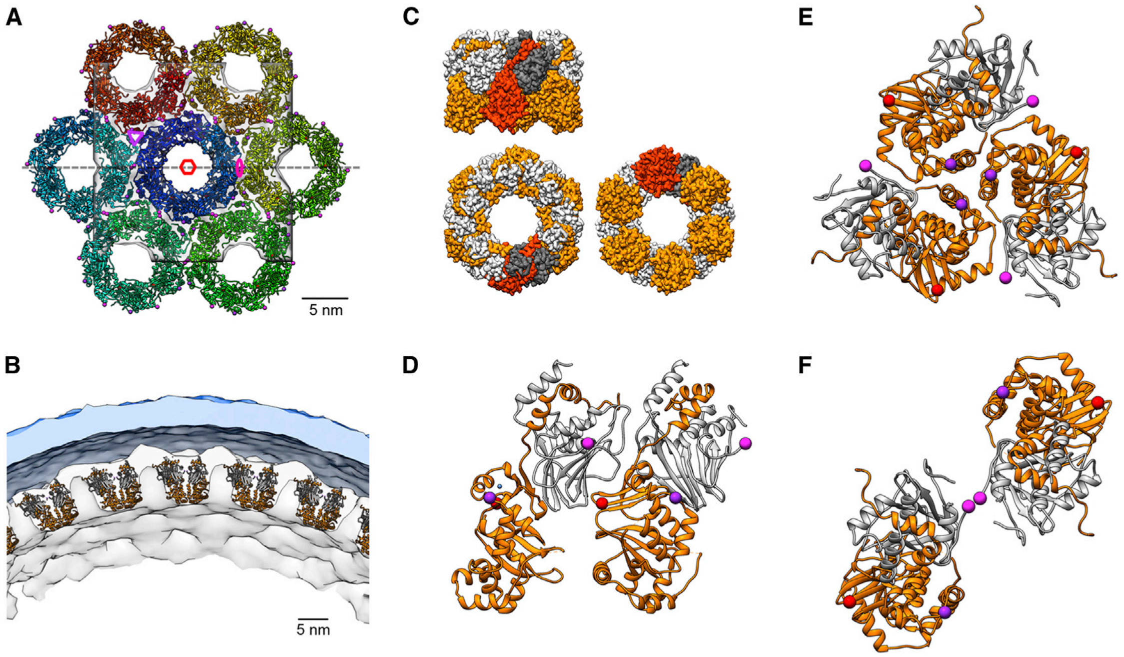

Figure 5. NEC Assembly to Curved Hexagonal Lattice

The pUL31-pUL34 complex was fitted into the cryo-EM map obtained from visualizing the precisely curved NEC lattice in situ (Hagen et al., 2015).

(A) Membrane-distal view (from vesicle interior) of the modeled hexameric lattice, consisting of 42 pUL31-pUL34 heterodimers arranged in 7 hexamers. The chains are rainbow colored. Dotted line depicts cutting planes used in (B). Red spheres indicate S77, magenta D22 and purple R101; cryoEM map in white.

(B) "Side" view of the modeled lattice, rotated 90° relative to (A) and shown at a lower magnification to show a larger area of the lattice. The pUL31 chains are in orange; pUL34 is in gray. The inner-nuclear-membrane-derived vesicle membrane is shown on top (light blue).

(C) A single hexamer of pUL31-pUL34 heterodimers. For one heterodimer, pUL31 is indicated in brick red, and pUL34 is indicated in dark gray, the remaining heterodimers are shown with pUL31 in orange and pUL34 in light gray. Top left: hexamer side view. Lower left: hexamer seen from the membrane. Lower right: hexamer seen from vesicle interior.

(D–F) Close-up views on the inter-heterodimeric interfaces formed in the hexametric lattice. Colors are as in (A), and the light blue sphere indicates the Zn ion. (D) Side view of the dimer-dimer interface within the hexamer, indicated by the purple oval in (A). (E) Top view of trimer interface between hexamers (same view as in A; hexamer is indicated by magenta triangle in A). (F) Top view of inter-hexameric dimer interactions (same view as in A; hexamer is indicated by red hexagon in A).

be interesting to identify alterations in the NEC that enable the curvature and the triggering event for this transition. From the extensive interactions within the heterodimer revealed here, it seems more likely that the relative angles between the rigid heterodimers, and between the heterodimers and the membrane, have to change for curvature formation, rather than the process being based on a hinge-like movement within the heterodimer. Finally, the structural analyses provided here give the blueprint of the necessary functional properties of cellular counterparts operational in the topologically related transport of large ribonucleoprotein particles (RNPs) through the nuclear envelope (Speese et al., 2012), for which the search is still ongoing.

EXPERIMENTAL PROCEDURES

NEC Expression, Purification, and Crystallization

DNA coding for PrV pUL31 (UniProt: G3G955), lacking the N-terminal 25 aa (aa 26–271), and truncated PrV pUL34 (UniProt: G3G8X8; aa 1–179) with C-terminal His6 tag were cloned into a pETDuet plasmid. The NEC was expressed in *E. coli* strain LEMO21(DE3) cultivated in a medium containing 50 nM ZnCl at 25°C; expression was induced with 1 mM isopropyl β-D-1-thiogalactopyranoside (IPTG). Expression of selenomethionine-labeled NEC was as described earlier but with 5 nM ZnCl. NEC was purified by, first, heat treatment followed by affinity chromatography and size exclusion chromatography; for details, see the Supplemental Experimental Procedures. The complex crystallized in 15.5%–17% polyethylene glycol (PEG) 6000, 1 M lithium chloride, 0.1 M L-malic acid, MES, and Tris buffer (pH 6.2–7.2).

Data Collection and Structure Determination

Diffraction data were recorded at the Diamond Light Source I04 beamline and processed with XIA2 (Kabsch, 2010). The structure was determined with the program PHENIX AUTOSOL (Adams et al., 2010), by single anomalous dispersion (SAD) phasing, using selenomethionine-labeled crystals. The model was built manually with the program COOT (Emsley and Cowtan, 2004) and restrained refinement (with TLS) with AUTOBUSTER (Bricogne, 2008). Final model geometry was checked with MolProbity (Davis et al., 2007). For data collection and refinement statistics, see Table S1.

Generation and Characterization of pUL31 and pUL34 Mutants by In Situ Cellular Studies

Localization and co-localization of NEC mutants were performed as described by Klupp et al. (2007). Functional complementation was as recently described by Paßvogel et al. (2015). For a brief description, see the Supplemental Experimental Procedures.

Fitting into EM Density

The NEC heterodimer was initially placed manually within the density of the sub-tomogram average (Hagen et al., 2015) constituting the central hexamer. A global rotational search (with a step size of ~12° and a translational search of 4 Å in steps of 2 Å in the radial plane) was then performed to produce ~140,000

different orientations for the heterodimer. Each reoriented heterodimer was symmetrized to produce a central hexamer; and, using a lattice spacing of 11.4 Å and a vesicle radius of 58 nm, six copies of the hexamer were modeled into the neighboring regions to produce a lattice. The presented model scored the highest using a combined score; for details, see the Supplemental Experimental Procedures.

ACCESSION NUMBERS

Atomic coordinates (PDB: 5E8C) as well as the hexagonal lattice model (PDB: 5FKI) were deposited to the PDB.

SUPPLEMENTAL INFORMATION

Supplemental Information includes Supplemental Experimental Procedures, five figures, two tables, and two movies and can be found with this article online at http://dx.doi.org/10.1016/j.celrep.2015.11.008.

AUTHOR CONTRIBUTIONS

T.Z., M.W., M.L., J.C., T.H., D.V., C.H., B.G.K., W.A., T.C.M., and K.G. intellectually conceived the experiments; T.Z., M.W., M.L., J.C., T.H., C.W., D.V., K.C.D., K.H., C.H., and B.G.K. performed the experiments; all authors processed and analyzed the data; and T.Z., C.H., W.A., T.C.M., and K.G. wrote the manuscript, and all authors commented on it.

ACKNOWLEDGMENTS

We are grateful to Natalia Silva Alves for superb technical support. This work was supported by a Wellcome Trust Senior Research Fellowship (090895/Z/09/Z) to K.G.; a Leverhulme Trust grant (RPG-2012-519) to K.G.; a German Research Foundation grant (DFG GR1990/2) to K.G.; a German Research Foundation grant (DFG Me 854/12-1) to T.C.M.; core funding of the Max Planck Society (to W.A.), a Wellcome Trust JIF award (060208/Z/00/Z) and a Wellcome Trust equipment grant (093305/Z/10/Z) to the Oxford Particle Imaging Centre; the Wellcome Trust core award (090532/Z/09/Z) to the Wellcome Trust Centre for Human Genetics; and a São Paulo Research Foundation Fellowship (FAPESP 2013/24972-1) to J.C.

Received: September 15, 2015
Revised: October 16, 2015
Accepted: October 31, 2015
Published: December 17, 2015

REFERENCES

Adams, P.D., Afonine, P.V., Bunkóczi, G., Chen, V.B., Davis, I.W., Echols, N., Headd, J.J., Hung, L.W., Kapral, G.J., Grosse-Kunstleve, R.W., et al. (2010). PHENIX: a comprehensive Python-based system for macromolecular structure solution. Acta Crystallogr. D Biol. Crystallogr. *66*, 213–221.

Bigalke, J.M., Heuser, T., Nicastro, D., and Heldwein, E.E. (2014). Membrane deformation and scission by the HSV-1 nuclear egress complex. Nat. Commun. *5*, 4131.

Bjerke, S.L., Cowan, J.M., Kerr, J.K., Reynolds, A.E., Baines, J.D., and Roller, R.J. (2003). Effects of charged cluster mutations on the function of herpes simplex virus type 1 U_L34 protein. J. Virol. *77*, 7601–7610.

Bricogne, G. (2008). Fourier transforms in crystallography: theory, algorithms and applications. In International Tables for Crystallography, Vol. B: Reciprocal Space, U. Shmueli, ed. (Dordrecht, the Netherlands: Springer), pp. 24–113.

Bubeck, A., Wagner, M., Ruzsics, Z., Lötzerich, M., Iglesias, M., Singh, I.R., and Koszinowski, U.H. (2004). Comprehensive mutational analysis of a herpesvirus gene in the viral genome context reveals a region essential for virus replication. J. Virol. *78*, 8026–8035.

Davis, I.W., Leaver-Fay, A., Chen, V.B., Block, J.N., Kapral, G.J., Wang, X., Murray, L.W., Arendall, W.B., 3rd, Snoeyink, J., Richardson, J.S., and Richardson, D.C. (2007). MolProbity: all-atom contacts and structure validation for proteins and nucleic acids. Nucleic Acids Res. *35*, W375–W383.

Emsley, P., and Cowtan, K. (2004). Coot: model-building tools for molecular graphics. Acta Crystallogr. D Biol. Crystallogr. *60*, 2126–2132.

Fuchs, W., Klupp, B.G., Granzow, H., Osterrieder, N., and Mettenleiter, T.C. (2002). The interacting U_L31 and U_L34 gene products of pseudorabies virus are involved in egress from the host-cell nucleus and represent components of primary enveloped but not mature virions. J. Virol. *76*, 364–378.

Hagen, C., Dent, K.C., Zeev-Ben-Mordehai, T., Grange, M., Bosse, J.B., Whittle, C., Klupp, B.G., Siebert, C.A., Vasishtan, D., Bäuerlein, F.J.B., et al. (2015). Structural basis of vesicle formation at the inner nuclear membrane. Cell *163*, Published online December 17, 2015. http://dx.doi.org/10.1016/j.cell.2015.11.029.

Johnson, D.C., and Baines, J.D. (2011). Herpesviruses remodel host membranes for virus egress. Nat. Rev. Microbiol. *9*, 382–394.

Kabsch, W. (2010). XDS. Acta Crystallogr. D Biol. Crystallogr. *66*, 125–132.

Klupp, B.G., Granzow, H., and Mettenleiter, T.C. (2000). Primary envelopment of pseudorabies virus at the nuclear membrane requires the U_L34 gene product. J. Virol. *74*, 10063–10073.

Klupp, B.G., Granzow, H., Fuchs, W., Keil, G.M., Finke, S., and Mettenleiter, T.C. (2007). Vesicle formation from the nuclear membrane is induced by coexpression of two conserved herpesvirus proteins. Proc. Natl. Acad. Sci. USA *104*, 7241–7246.

Kochańczyk, T., Drozd, A., and Krężel, A. (2015). Relationship between the architecture of zinc coordination and zinc binding affinity in proteins–insights into zinc regulation. Metallomics *7*, 244–257.

Leigh, K.E., Sharma, M., Mansueto, M.S., Boeszoermenyi, A., Filman, D.J., Hogle, J.M., Wagner, G., Coen, D.M., and Arthanari, H. (2015). Structure of a herpesvirus nuclear egress complex subunit reveals an interaction groove that is essential for viral replication. Proc. Natl. Acad. Sci. USA *112*, 9010–9015.

Lorenz, M., Vollmer, B., Unsay, J.D., Klupp, B.G., García-Sáez, A.J., Mettenleiter, T.C., and Antonin, W. (2015). A single herpesvirus protein can mediate vesicle formation in the nuclear envelope. J. Biol. Chem. *290*, 6962–6974.

Lötzerich, M., Ruzsics, Z., and Koszinowski, U.H. (2006). Functional domains of murine cytomegalovirus nuclear egress protein M53/p38. J. Virol. *80*, 73–84.

McMahon, H.T., and Gallop, J.L. (2005). Membrane curvature and mechanisms of dynamic cell membrane remodelling. Nature *438*, 590–596.

Mettenleiter, T.C., Müller, F., Granzow, H., and Klupp, B.G. (2013). The way out: what we know and do not know about herpesvirus nuclear egress. Cell. Microbiol. *15*, 170–178.

Paßvogel, L., Klupp, B.G., Granzow, H., Fuchs, W., and Mettenleiter, T.C. (2015). Functional characterization of nuclear trafficking signals in pseudorabies virus pU_L31. J. Virol. *89*, 2002–2012.

Reynolds, A.E., Ryckman, B.J., Baines, J.D., Zhou, Y., Liang, L., and Roller, R.J. (2001). $U_{(L)}31$ and $U_{(L)}34$ proteins of herpes simplex virus type 1 form a complex that accumulates at the nuclear rim and is required for envelopment of nucleocapsids. J. Virol. *75*, 8803–8817.

Roller, R.J., Bjerke, S.L., Haugo, A.C., and Hanson, S. (2010). Analysis of a charge cluster mutation of herpes simplex virus type 1 U_L34 and its extragenic suppressor suggests a novel interaction between pU_L34 and pU_L31 that is necessary for membrane curvature around capsids. J. Virol. *84*, 3921–3934.

Speese, S.D., Ashley, J., Jokhi, V., Nunnari, J., Barria, R., Li, Y., Ataman, B., Koon, A., Chang, Y.T., Li, Q., et al. (2012). Nuclear envelope budding enables large ribonucleoprotein particle export during synaptic Wnt signaling. Cell *149*, 832–846.

Votteler, J., and Sundquist, W.I. (2013). Virus budding and the ESCRT pathway. Cell Host Microbe *14*, 232–241.

 ©2015 The Authors

Cell Reports
Supplemental Information

Crystal Structure of the Herpesvirus Nuclear Egress Complex Provides Insights into Inner Nuclear Membrane Remodeling

Tzviya Zeev-Ben-Mordehai, Marion Weberruß, Michael Lorenz, Juliana Cheleski, Teresa Hellberg, Cathy Whittle, Kamel El Omari, Daven Vasishtan, Kyle C. Dent, Karl Harlos, Kati Franzke, Christoph Hagen, Barbara G. Klupp, Wolfram Antonin, Thomas C. Mettenleiter, and Kay Grünewald

SUPPLEMENTAL FIGURES

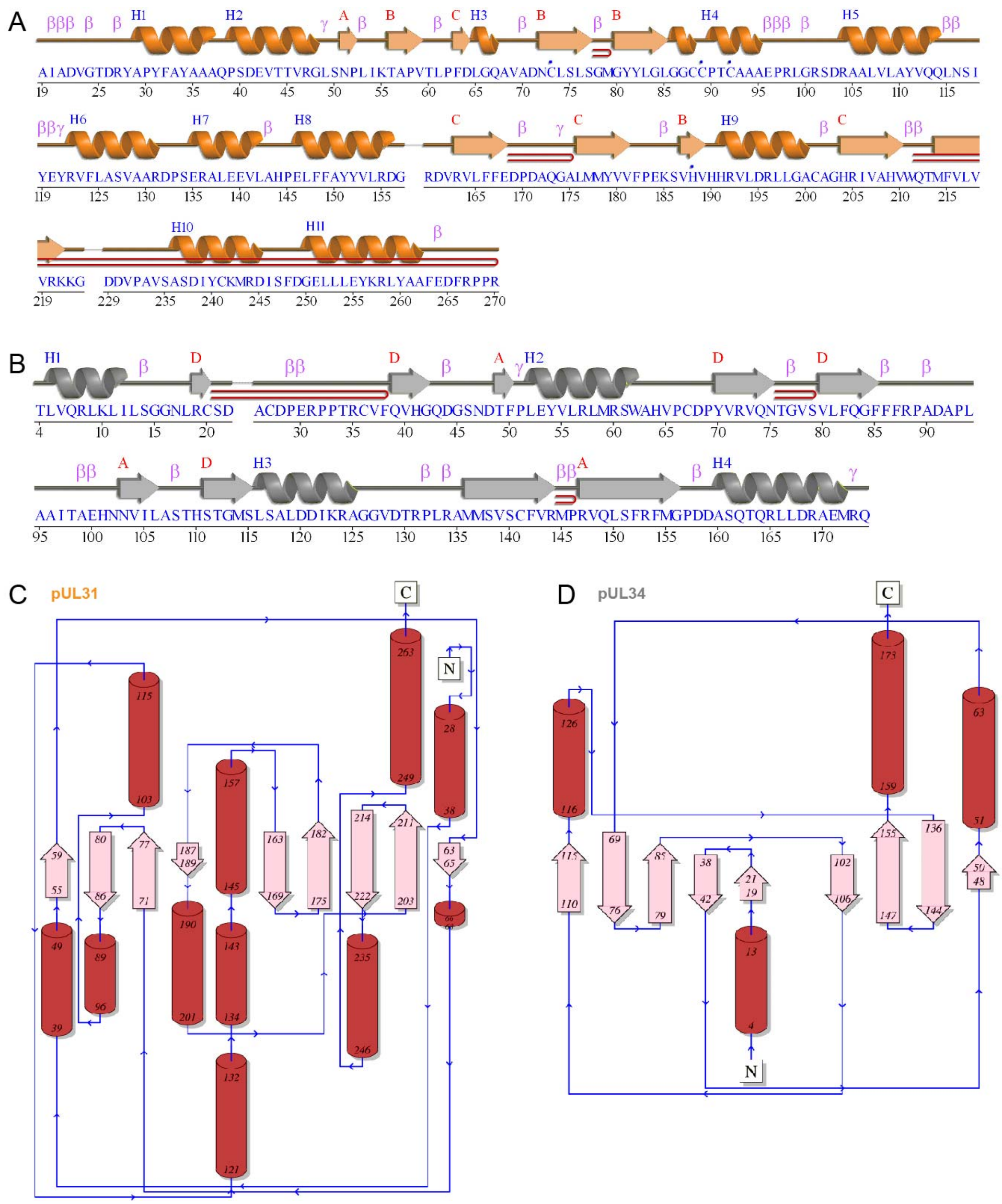

Figure S1. Secondary structure elements and topology mapping, related to Figure 1.
(A) and (B) Secondary structure elements mapped onto the sequence of pUL31 and pUL34, respectively. (C) and (D) topology maps of pUL31 and pUL34, respectively.

A

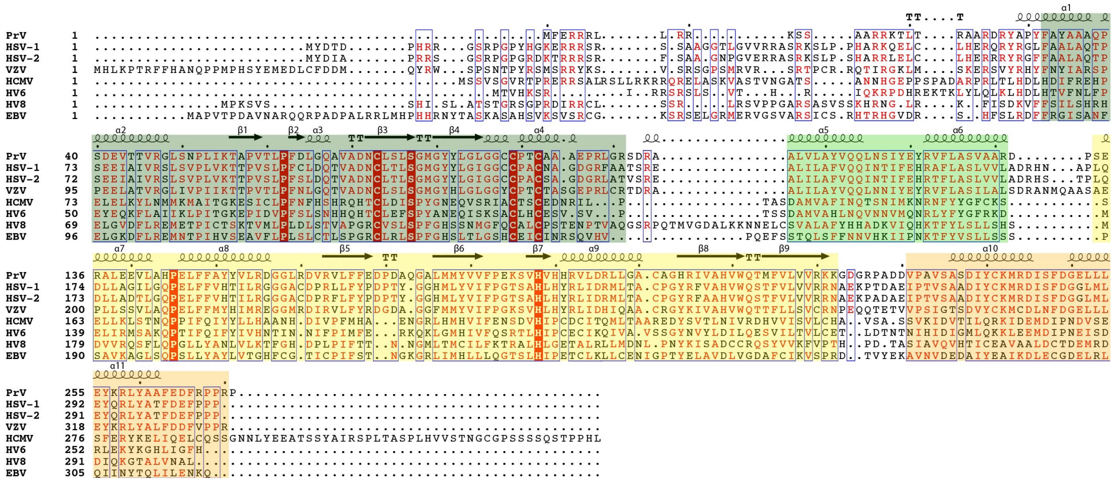

B

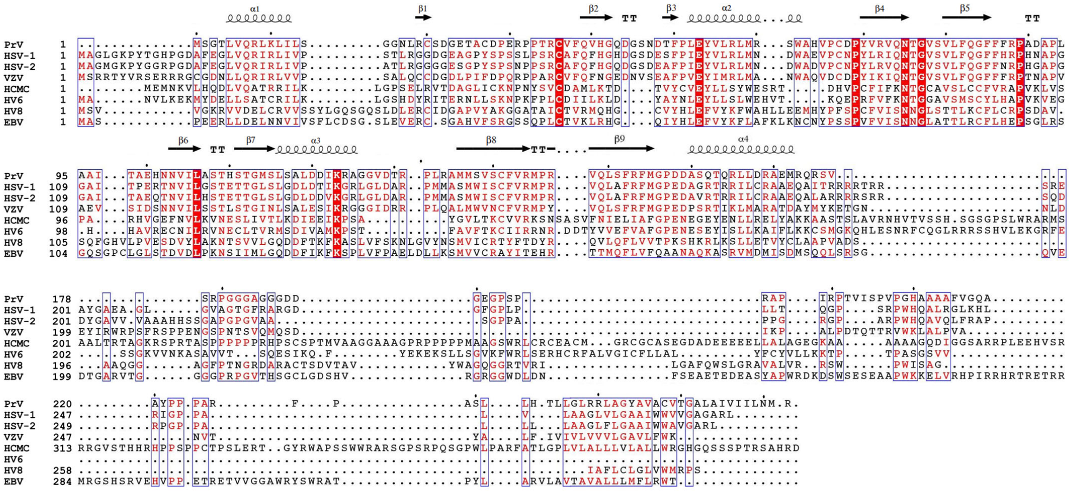

Figure S2. Multiple sequence alignment with selected homologs, related to Figure 1.
(A) Alignment for pUL31. The alignment includes pseudorabies virus (PrV; accession G3G955), human herpes simplex virus 1 (HSV1; accession P10215), human herpes simplex virus 2 (HSV2; accession P89454), human herpesvirus 3/ Varicella-zoster virus (VZV; accession P09283), human herpesvirus 5/ human cytomegalovirus (HCMV M53; accession F5HFZ4), human herpesvirus 6A/ human B lymphotropic virus (accession P28865), Human herpesvirus 8 type P (HV6; accession F5H982) and Epstein-Barr virus/ human herpesvirus 4 (HV4; accession P0CK48). Highly conserved residues are shaded and boxed. The four conserved regions CR1-CR4 1 (Lötzerich et al., 2006) are coloured: CR1, dark green; CR2, light green; CR3, yellow and CR4, orange.
(B) Alignment for pUL34. The alignment includes pseudorabies virus (PrV; accession G3G8GX), human herpes simplex virus 1 (HSV-1; accession P1021), human herpes simplex virus 2 (HSV-2; accession P89457), human herpesvirus 3/ Varicella-zoster virus (VZV; accession P09280), human herpesvirus 5/ human cytomegalovirus (HCMV M50; accession Q6SW81), human herpesvirus 6A/ human B lymphotropic virus (HV6; accession P52465), Human herpesvirus 8 type P (HV8; accession F5HA27) and Epstein-Barr virus/ human herpesvirus 4 (HV4; accession P03185).
The secondary structure elements of PrV virus are shown above the sequence, coloured and labelled per domain, every tenth amino acid is marked with a dot. Highly conserved residues are shaded and boxed. Clustal Omega (Sievers et al., 2011) and ESPript server (Robert and Gouet, 2014) were used for the alignment of representative herpesvirus homologs.

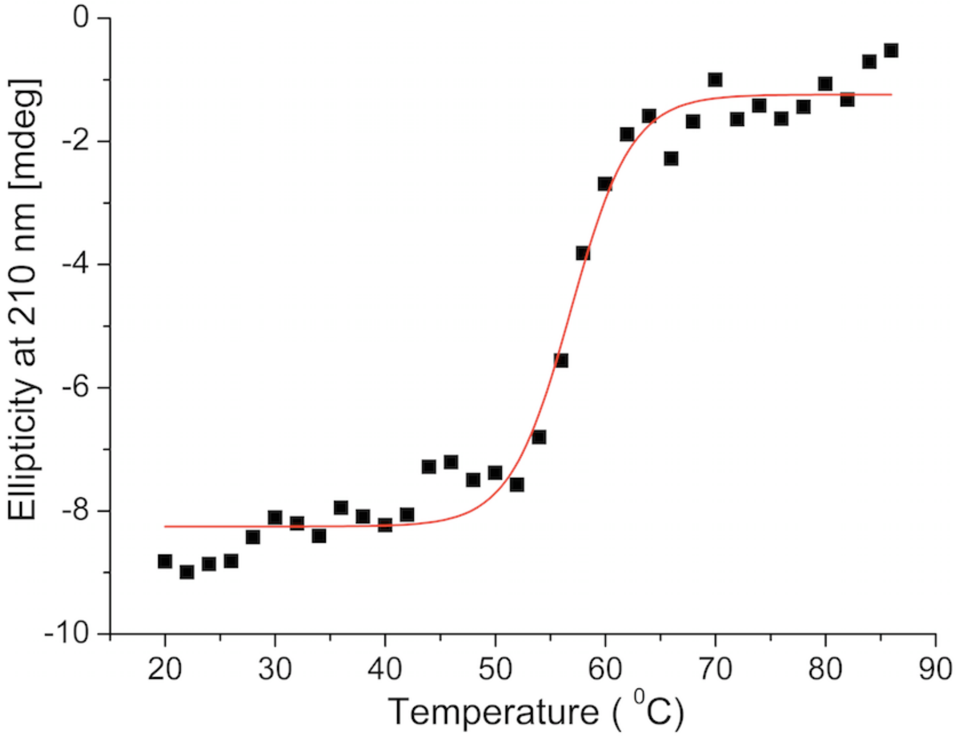

Figure S3. CD thermal denaturation profiles, related to Figure 3.
Thermal denaturation profiles of the NEC were recorded by circular dichroism. Melting temperature (Tm) is 57 °C. The denaturation is irreversible.

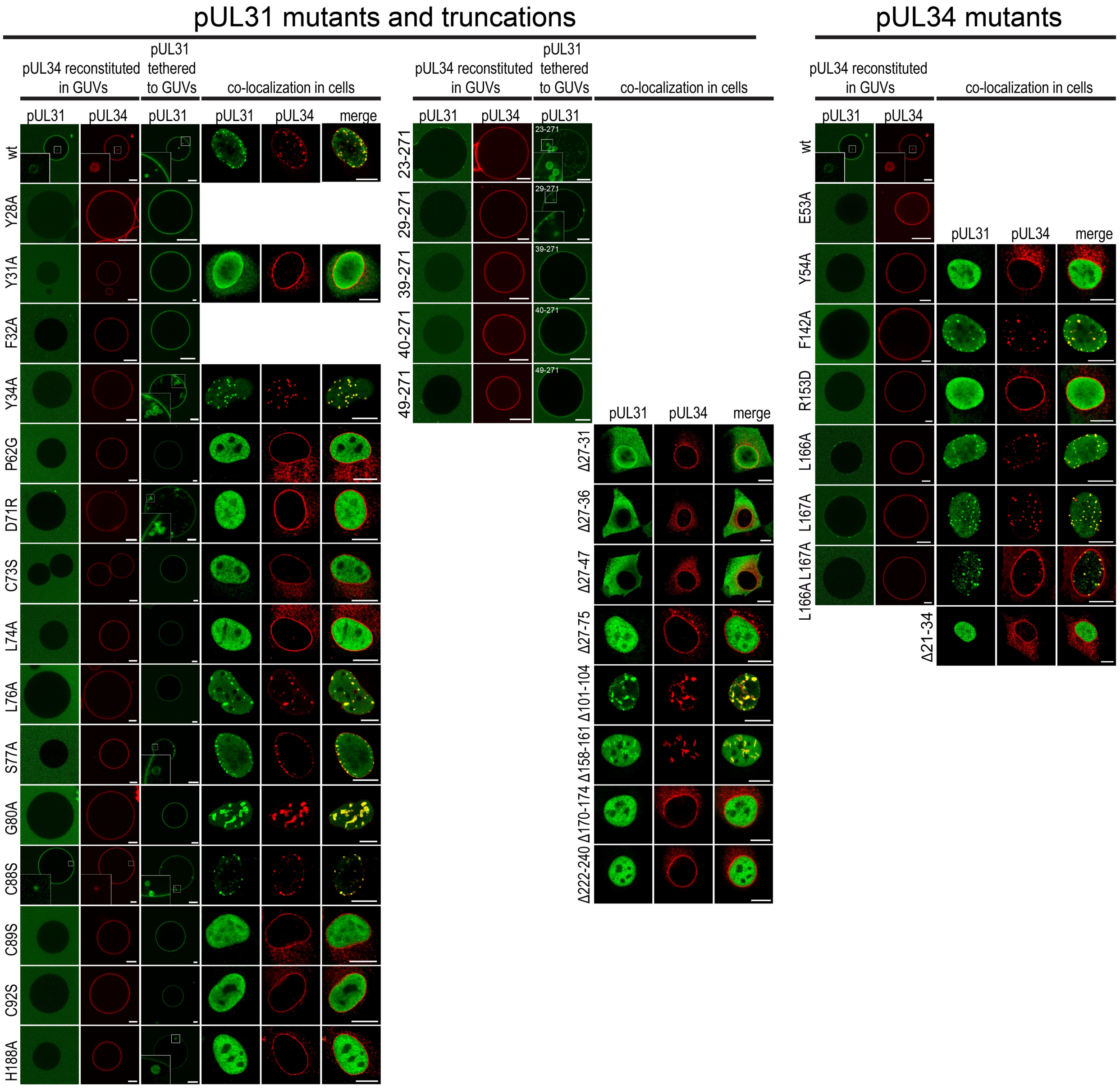

Figure S4. Summary of *in situ* and *in vitro* phenotypes of all reported mutations, related to Table S2, Figures 2 and 3.
pUL34 reconstituted in GUVs: Analysis of *in vitro* vesicle formation ability of pUL31 mutant on pUL34 giant unilamellar vesicles (GUVs). GUVs with reconstituted Alexa-546 labelled wildtype pUL34 (red channel) were incubated with EGFP-pUL31 wildtype or mutant proteins or EGFP (green channel). Binding of EGFP-pUL31 to pUL34 GUVs and intra-GUV vesicle formation were examined by confocal microscopy. Scale bars: 10 µm.
pUL31 tethered to GUVs: Analysis of *in vitro* vesicle formation ability of pUL31 mutants. His_6-EGFP, His_6-EGFP-pUL31 or His_6-EGFP-pUL31 mutants were directly tethered to GUVs containing Ni-NTA-DGS and vesicle formation was analysed by confocal microscopy. Scale bars: 10 µm.
Co-localization in cells: Rabbit kidney (RK13) cells were stably transfected with wildtype or mutant pUL31 encoding constructs (green channel) with wildtype pUL34 (red channel). Proteins were detected by immunofluorescence with respective antibodies followed by confocal microscopy. Scale bars: 10 µm.
Mutants are marked on the left of each raw.

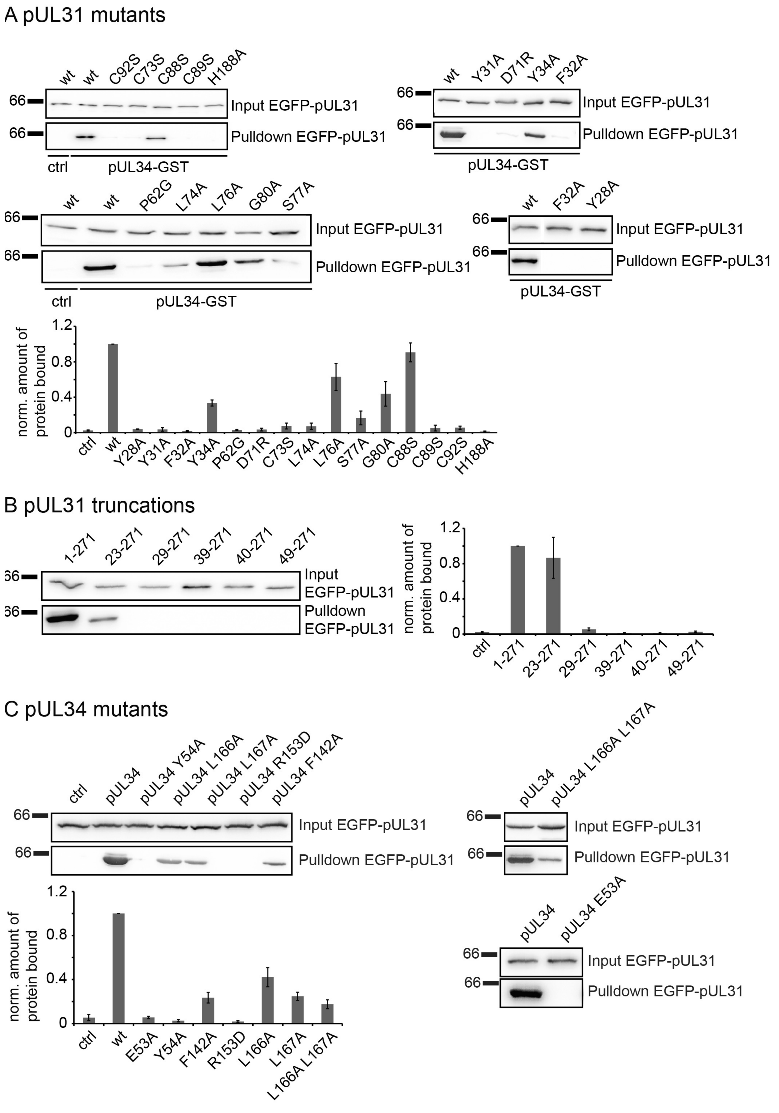

Figure S5. GST-pull-down assays for pUL31 and pUL34 mutants, related to Tables S2, Figures 2 and 3. Glutathion-S-Transferase (GST)-pull-downs of pUL31 mutants (A) and truncations (B). GST-pull-down experiments were performed using GST (ctrl) or GST-pUL34 (aa 1-240, TMR) as baits and EGFP-pUL31 *wt* and mutants as prey. GST-pUL34-bound EGFP-pUL31 was eluted with TEV protease containing buffer which cleaves the GST-fusions C-terminal of the GST tag. Input and eluate were analysed by western blotting using anti-EGFP-antibodies. Pull-downs of three independent experiments were quantified and the amount of eluted mutant pUL31 is shown normalized to *wt* pUL31. Error bars: SEM (C) GST-pull-downs of pUL34 mutants. GST-pull-down experiments were performed using GST (ctrl), GST-pUL34 (aa 1-240, TMR) or the respective mutants as baits. *wt* EGFP-pUL31 served as prey. GST-pUL34-bound EGFP-pUL31 was eluted with TEV protease containing buffer. Input and eluate were analysed by Western blotting using anti-EGFP-antibodies. Pull-downs of three independent experiments were quantified and the amount of eluted *wt* pUL31 bound to pUL34 versions is shown normalized to *wt* pUL34. Error bars: SEM

Table S1. Table of data collection and structure refinement statistics, related to Figure 1

**Data collection**	
Beamline	DLS-I04
Space group	$P\,4_3\,2\,2$
Unit cell parameters (Å,°)	a=b=91.69, c=108.26<br>α=β=γ=90
No. of crystals	5
Wavelength	0.9790
Resolution (Å)	70.0-2.9 (2.98-2.90)
No. of unique reflections	10771 (777)
Completeness (%)	99.9 (99.9)
Multiplicity	227.3 (209.1)
Anomalous completeness	99.9 (99.9)
Anomalous multiplicity	125.3 (111.6)
I/σ(I)	35.6 (4.2)
R_{merge} (%)	30.8 (346.6)
R_{pim} (%)	2.0 (23.8)
CC half	1.0 (0.93)
**Refinement**	
Resolution (Å)	50.0-2.9
R_{work}/R_{free} (%)	21.9/26.5
R.m.s.d. bond (Å)	0.007
R.m.s.d. angle (º)	0.90
Mean B-factors/Wilson plot ($Å^2$)	92/87
Number of atoms	3175
Metal (Zn)	1
Total number of residues	406
Ramachandran plot (%)<br>favoured/allowed/outliers	95.7/100/0

Table S2. **Phenotypes of mutants analyzed, related to Figure 2 and Figure S4**

Position	Vesicle formation on pUL34 GUV*	Vesicle formation if directly tethered**	pUL31 binding to pUL34 in GST-pulldowns	Localization in transfected cells	Colocalization pUL31-pUL34 in co-transfected cells	Complementa-tion of PrV-DUL31	Electron microscopy analysis of complementation assays
**in pUL31**							
wt	pos.	pos.	100%	nucleus	pos.	pos.	all stages of virion maturation (Fuchs et al., 2002)
Y28A	neg.	neg.	0%				
Y31A	neg.	strongly reduced	0%	nucleus	neg.	neg.	
F32A	neg.	neg.	0%				
Y34A	neg.	pos.	30%	nucleus	pos.	reduced	
P62G	neg.	neg.	0%	nucleus	neg.	neg.	Capsids dispersed in nucleus
D71R	neg.	pos.	0%	nucleus	neg.	neg.	
C73S	neg.	neg.	10%	nucleus	neg.	neg.	Capsids dispersed in nucleus
L74A	neg.	neg.	10%	nucleus	neg.	neg.	Capsids dispersed in nucleus
L76A	neg.	neg.	60%	nucleus	pos.	neg.	Large infoldings of NM; capsids at INM; no budding
S77A	neg.	reduced	20%	nucleus	pos.	neg.	Capsids at INM; no budding
G80A	neg.	neg.	40%	nucleus	pos.	neg.	Large infoldings of NM; capsids at INM; no budding
C88S	pos.	pos.	90%	nucleus	pos.	pos.	all stages of virion maturation
C89S	neg.	neg.	10%	nucleus	neg.	neg.	Capsids dispersed in nucleus
C92S	neg.	neg.	10%	nucleus	neg.	neg.	Capsids dispersed in nucleus
H188A	neg.	reduced	0%	nucleus	neg.	neg.	Capsids dispersed in nucleus
aa23-271	reduced	reduced	90%				
aa29-271	neg.	reduced	10%				
aa39-271	neg.	strongly reduced	0%				
aa40-271	neg.	neg.	0%				
aa49-271	neg.	neg.	0%				
CR1 Δ5 (Δ27-31)				nucleus	neg.	neg.	

CR1 Δ10<br>(Δ27-36)				nucleus +<br>cytoplasm	neg.	neg.	
CR1 Δ20<br>(Δ27-47)				nucleus +<br>cytoplasm	neg.	neg.	
CR1 Δ50<br>(Δ27-75)				nucleus +<br>cytoplasm	neg.	neg.	
Δ101-104				nucleus	pos.	neg.	
Δ158-161				nucleus	pos.	neg.	
Δ170-174				nucleus	neg.	neg.	
Δ222-240				nucleus	neg.	neg.	
**in pUL34**							
wt	pos.		100%	rim	pos.	pos.	all stages of virion maturation (Klupp et al., 2000)
E53A	neg.		10%				
Y54A	neg.		0%	rim	neg.	neg.	
E53AY54A				rim	neg.	neg.	Capsids dispersed in nucleus
F142A	neg.		20%	rim	pos.	reduced	
R153D	neg.		0%	rim	neg.	neg.	
L166A	reduced		40%	rim	pos.	pos.	
L167A	reduced		20%	rim	pos.	reduced	
L166AL167A	neg.		20%	rim	pos.	neg.	Capsids dispersed in nucleus
Δ2-4 (N5)				rim	pos.	pos.	
Δ2-9 (N10)				rim	neg.	neg.	
Δ21-34				rim + ER	neg.	(unstable)	

* induction of vesicles by pUL31 on GUVs with reconstituted pUL34 (pos.: pUL31 binding to the GUV membranes and induction of intra-GUV vesicles identical to wildtype level; reduced: only weak pUL31 binding to pUL34-GUVs, intra-GUV vesicles rare; neg.: no pUL31 binding to pUL34-GUVs, no vesicle induction).

** Induction of intra-GUV vesicles if pUL31 is directly tethered to the GUV membrane (pos.: number of vesicle containing GUVs 80-100% of wildtype level; reduced: number of vesicle containing GUVs 40-80% of wildtype level; strongly reduced: number of vesicle containing GUVs 10-40% of wildtype level; negative: no vesicle induction)

SUPPLEMENTAL MOVIE LEGENDS

Movie S1. pUL31 surface electrostatics, related to Figure 1.

Movie S2. pUL34 surface electrostatics, related to Figure 1.

SUPPLEMENTAL EXPERIMENTAL PROCEDURES

Purification and Crystallization

NEC was purified by first heat treatment (42 °C for 15 minutes) followed by clarification (25,000*g*, 15 min, 16 °C). Next the complex was purified by affinity chromatography (Co^{2+}-Sepharose 6-fast flow) and size exclusion chromatography in 10 mM Tris-HCl pH7.4, 75 mM NaCl, 3 mM DTT and 0.1mM TCEP. The complex was concentrated to ~15 mg/ml and 5 mM TCEP-HCL was added before crystallisation. Crystals were flash frozen using 25 % (v/v) glycerol/reservoir solution as cryo-protectant.

Circular dichroism spectroscopy

Circular dichroism (CD) measurements were performed on a JASCO J-815 CD spectropolarimeter (JASCO Corporation, Hachioji-shi, Tokyo, Japan). Denaturation of NEC were monitored at 210 nm in the temperature range 20–85 °C with a concentration of 0.1 mg/ml, scanning speed of 50 nm/min, 10 mM phosphate buffer, 20 mM NaCl, pH 7.4. Data analysis was carried out using Origin 7.0. The thermal denaturation data were fit to a derivation of the Boltzmann equation for the two-state unfolding model using Origin 7.0 to obtain the midpoint of denaturation (the thermal melting point).

GUV *in vitro* mutant characterisation

Details are described in (Lorenz et al., 2015). In brief, constructs for expression of PrV pUL31 and pUL34 were generated from a synthetic DNA optimized for codon usage in *E. coli* (Geneart) and pUL31 mutants were generated on this basis using the Quick change mutagenesis kit (Agilent). pUL31 and pUL34 proteins were expressed and purified, GUVs generated and GST pull-down assays analysed as described (Lorenz et al., 2015).
Alexa Fluor 546 carboxylic acid succinimidyl ester was obtained from Life Technologies and detergents from Calbiochem. The nuclear envelope lipid mix consists of 5 mol % cholesterol, 2.5 mol % sphingomyeline, 2.5 mol % Na-phosphatidylserine, 10 mol % Na-phosphatidylinositol, 20 mol % phosphatidylethanolamine, and 60 mol % phosphatidylcholine (all from Avanti Polar Lipids) .

Generation and characterization of pUL31 and pUL34 mutants by cellular studies *in situ*

Site directed mutagenesis was used to generate single amino acid substitutions as well as internal deletion mutants by modifying either pcDNA-UL31 (Fuchs et al., 2002) or pcDNA-UL34 (Klupp et al., 2000) using the QuickChange II XL site-directed mutagenesis kit (Agilent Technologies) as described recently (Paßvogel et al., 2013).
To test for localization of the mutated proteins rabbit kidney (RK13) cells were transfected with the corresponding expression plasmids by calcium phosphate co-precipitation (Graham and van der Eb, 1973). Transfected cells were fixed with 3 % paraformaldehyde / 0.3 % TritonX100 two days after transfection and labelled either with the polyclonal rabbit anti-pUL31 (Fuchs et al., 2002) or the polyclonal rabbit anti-pUL34 serum (Klupp et al., 2000), which were diluted 1:500 in PBS. After incubation at room temperature for 1 h, bound antibody was detected with Alexa Fluor 488 goat anti-rabbit antibodies (Invitrogen).
For co-localization studies, RK13 cells were co-transfected with the mutated plasmids and the corresponding plasmid expressing the wild-type complex partner. To simultaneously detect pUL31 and pUL34, transfected cells were fixed as described above and incubated with rabbit polyclonal anti-pUL31 and mouse polyclonal anti-pUL34 antisera (Klupp et al., 2007) Alexa Fluor 488 anti-rabbit and Alexa Fluor 555 anti-mouse antibodies (Invitrogen) were applied to detect bound anti-pUL31 and anti-pUL34 antibodies, respectively. All secondary antibodies were diluted 1:1,000 in PBS. Fluorescence images were recorded with a laser scanning confocal microscope (SP5; Leica, Mannheim, Germany).
Cell lines stably expressing mutant pUL31 or pUL34 were generated after transfection of RK13 cells with the corresponding expression plasmids. Two days post transfection cells were split and incubated with G418 (0.5 µg/µl) containing medium to select for transfected cells. After 12 to 14 days cell colonies were visible and picked by aspiration. Single cell clones were tested for pUL31 or pUL34 expression by indirect immunofluorescence and by western blotting using the corresponding monospecific rabbit sera as described above. Cell clones efficiently expressing the mutated proteins were propagated and used for single step growth kinetics.
To test for functional complementation RK13 cells expressing wild-type or mutated pUL31 or pUL34 were infected with PrV-*wt* (PrV strain Kaplan; Kaplan and Vatter (1959)) and the corresponding deletion mutants PrV-ΔUL31 (Fuchs et al., 2002) or PrV-ΔUL34 (Klupp et al., 2000) with a multiplicity of infection of 5. 24h post infection cells and supernatant were harvested and titrated on RK13-UL31 or RK13-UL34 cells, respectively. One-step growth assays were repeated three times and mean values were calculated. Results were ranked as follows: Titres comparable to PrV-*wt* from the corresponding cell line or at maximum 10-fold reduced as positive, titres reduced between 10- and 50-fold as reduced, titres lower than 50-fold but more than 10-fold than those from non-complementing cells as strongly reduced, and titres equalling those of the respective deletion mutant on RK13 cells (negative control) as negative.
To investigate the defect in greater detail, mutant pUL31 or pUL34 expressing cells were infected with the corresponding deletion mutant at a multiplicity of infection of 1. Cells were fixed and processed for transmission electron microscopy 14 hours post infection as described previously (Klupp et al., 2000).

Scores used for the fitting into EM density

To determine which model best fit the map, two scores were used. First was a 'protrusion score' that measured the percentage of backbone atoms of a single heterodimer that were placed in voxels with intensity less than 2 (*i.e.* sitting outside the envelope of the map). Second was a clash score, which measured the percentage of all atoms of a single heterodimer that were within 1.2 Å of an atom from a neighbouring heterodimer. These scores were summed and the models ranked accordingly. The model shown here had the lowest combined protrusion and clash score.
